# Mobilization of science advice by the Canadian federal government to support the COVID-19 pandemic response

**DOI:** 10.1057/s41599-023-01501-8

**Published:** 2023-01-17

**Authors:** Dominika Bhatia, Sara Allin, Erica Di Ruggiero

**Affiliations:** 1 North American Observatory on Health Systems and Policies, Toronto, ON Canada; 2 Women’s College Research Institute, Women’s College Hospital, Toronto, ON Canada; 3 Institute of Health Policy, Management and Evaluation, Dalla Lana School of Public Health, University of Toronto, Toronto, ON Canada; 4 Social and Behavioural Health Sciences Division, Dalla Lana School of Public Health, University of Toronto, Toronto, ON Canada; 5 Centre for Global Health, Dalla Lana School of Public Health, University of Toronto, Toronto, ON Canada

**Keywords:** Science, technology and society, Social policy

## Abstract

The procurement and provision of expert-driven, evidence-informed, and independent science advice is integral to timely decision-making during public health emergencies. The 2019 coronavirus disease (COVID-19) pandemic has underscored the need for sound evidence in public health policy and exposed the challenges facing government science advisory mechanisms. This paper is a jurisdictional case study describing (i) the federal science advice bodies and mechanisms for public health in Canada (i.e., the federal science advice “ecosystem”); and (ii) how these bodies and mechanisms have mobilized and evolved to procure expertise and evidence to inform decisions during the first two years of the COVID-19 pandemic. We reviewed publicly accessible Government of Canada documents, technical reports, and peer-reviewed articles available up to December 2021. Canada’s federal landscape of science advisory bodies for public health within the Health Portfolio was largely shaped by Canada’s experiences with the 2003 severe acute respiratory syndrome and 2009 H1N1 outbreaks. In parallel, Canada has a designated science advisory apparatus that has seen frequent reforms since the early 2000s, with the current Office of the Chief Science Advisor created within the Science Portfolio in 2018. The COVID-19 pandemic has further complicated Canada’s science advice ecosystem, with involvement from departments, expert advisory groups, and partnerships within both the federal Health and Science Portfolios. Although the engagement of federal departments outside the health sector is promising, the COVID-19 experience in Canada supports the need to institutionalize science advisory bodies for public health to improve pandemic preparedness and ensure rapid mobilization of well-coordinated and independent advice in future emergencies. This review also identified pressing areas for further inquiry to strengthen science advice for public health in Canada, including to assess the independence of science advisory actors and the interaction between federal and subnational authorities.

## Introduction

The coronavirus disease 2019 (COVID-19) pandemic has placed significant pressures on the science-policy interface due to its rapidly changing trajectory, considerable health, social, and economic impacts, and constantly evolving evidence base (Allin et al., 2021). The World Health Organization (WHO) declared COVID-19 a public health emergency of international concern on January 30, 2020 and a global pandemic on March 11, 2020 (World Health Organization, 2020). In Canada, the first case of COVID-19 was confirmed on January 25, 2020 in a traveler from Wuhan, China (Public Health Agency of Canada, 2020b). As of December 2021, Canada entered a fifth wave of the disease, with a total of nearly 2 million cases and over 30 thousand deaths (Public Health Agency of Canada, 2020b, 2021k). While Canada’s overall COVID-19 mortality rate has been lower than that of other comparable nations, such as the United States (US) and the United Kingdom (UK) (Our World in Data, 2022; Unruh et al., 2021), much of the burden of COVID-19 infections and deaths has been borne by low income, racialized, migrant, and long-term care populations (Canadian Institute for Health Information, 2020; Guttmann et al., 2020; Public Health Agency of Canada, 2021k). Similar to other countries, Canada has relied on different public health measures to mitigate the spread of COVID-19, including state of emergency declarations, border closures, physical distancing guidelines, movement and gathering restrictions, school and workplace closures, and mask mandates (Allin et al., 2021; McCoy et al., 2020; Public Health Agency of Canada, 2021k; Unruh et al., 2021). Public vaccination efforts began in December 2020, with 83% of eligible individuals (aged ≥5 years) having received at least one dose and 77% at least two doses of an approved COVID-19 vaccine in Canada by December 2021 (Government of Canada, 2022). The administration of a third vaccine dose among adults began in December 2021 (Government of Canada, 2022).

Canada’s federal science advice landscape for public health emergencies was shaped by two infectious disease outbreaks. In 2003, Canada had the greatest burden of the severe acute respiratory syndrome (SARS) cases than any other country outside of Asia, where the virus was initially identified (Naylor, 2003). Between February and July of 2003, Canada experienced two waves of the disease across two provinces (Ontario and British Columbia) (Naylor, 2003). The SARS outbreak triggered major public health reforms at the federal level, including the creation of a national public health agency in 2004, the introduction of a new mechanism to coordinate public health response across the federal and subnational^1^ governments, and the release of pandemic preparedness and response plans (Naylor, 2003). The ensuing two waves of the H1N1 influenza virus between April 2009 and January 2010 presented the first test of these structures (Eggleton et al., 2010; Pan-Canadian Public Health Network, 2018a; Public Health Agency of Canada, 2011). Post-H1N1 government inquiries revealed that similar issues persisted from SARS through to the H1N1 outbreak, including the availability of public health expertise, surge capacity, and rapid evidence to inform government decisions and guidance (Canadian Public Health Association, 2021; Eggleton et al., 2010; Naylor, 2003; Public Health Agency of Canada, 2011).

The science advice “ecosystem” plays a crucial role in supporting the management of pandemic emergencies, which require timely government decisions carrying significant population impacts in the context of uncertainty. Despite accumulating knowledge about the virus over the first two years of the pandemic, COVID-19 remains a whole-of-society crisis necessitating multidisciplinary expertise and evidence on issues ranging from the effectiveness of clinical therapeutics and vaccines to public uptake of protective public health measures amid disease resurgences driven by novel viral variants. As one of the most decentralized federations among high-income countries, Canada presents an interesting case study of mobilizing science advice in a pandemic emergency, since the development of technical guidance is largely within the remit of the federal government^2^, while the implementation of public health measures is primarily the responsibility of subnational governments. In this paper, we identify opportunities for strengthening federal science advice for public health emergencies in Canada. To do so, we describe (i) Canada’s federal science advice ecosystem established before the COVID-19 pandemic (the pandemic “playbook”); and (ii) how Canada’s federal science advice ecosystem has evolved and mobilized in the first two years of the COVID-19 pandemic.

## Methods

We conducted a jurisdictional descriptive case study of Canada’s federal science advice ecosystem, reliant on a literature review of publicly accessible primary (i.e., Government of Canada technical reports and guidance) and secondary (i.e., peer-reviewed literature) documents. Study methodology is detailed in Supplementary file 1, including concept definitions, document retrieval processes, and synthesis approaches. Briefly, the document search, using iterative snowballing techniques, focused on the period between the 2003 SARS outbreak in Canada and December 2021. Local public health experts were contacted to verify the completeness of the retrieved information. We identified key advisory bodies in the federal science advice ecosystem, including those involved in evidence generation, brokerage, communication, and decision-making. We considered advisory bodies to be part of the federal science advice ecosystem if evidence that the body has an advisory relationship with the federal government could be established in the literature.

The manuscript findings are organized as follows: first, we document the advisory bodies that constituted the federal science advice ecosystem before the COVID-19 pandemic; then, we discuss how these bodies have been mobilized to inform the COVID-19 response, highlighting new science advisory bodies created for convening experts and generating evidence to inform federal decision-making. In the discussion section, we reflect on the mobilization and evolution of Canada’s federal science advice ecosystem during the first two years of the COVID-19 pandemic in the context of the broader literature on science advice to provide policy and research recommendations. This study was conducted as part of the Evaluation of Science Advice in a Pandemic Emergency (EScAPE) international case series. Box 1 presents the full list of acronyms used in this paper.

Box 1 List of acronyms used

**Table:** 

CanCOGeN	Canadian COVID-19 Genomics Network	CPIP	Canadian Pandemic Influenza Preparedness	OCSA	Office of the Chief Science Advisor
CCA	Council of Canadian Academies	DSA	Departmental Science Advisors	PHAC	Public Health Agency of Canada
CIHR	Canadian Institutes of Health Research	FPT	Federal, provincial, and territorial	PHN	Pan-Canadian Public Health Network
CITF	COVID-19 Immunity Task Force	HPOC	Health Portfolio Operations Centre	SAC	Special Advisory Committee
CMOH	Chief Medical Officer of Health	ISED	Innovation, Science and Economic Development	SARS	Severe acute respiratory syndrome
COI	Conflict of interest	LAC	Logistics Advisory Committee	SSHRC	Social Sciences and Humanities Research Council of Canada
CoVaRR-Net	Coronavirus Variants Rapid Response Network	NACI	National Advisory Committee on Immunization	STIC	Science, Technology and Innovation Council
COVID-19	Coronavirus disease 2019	NCC	National Collaborating Centres	TAC	Technical Advisory Committee
COVID-END	COVID-19 Evidence Network to Support Decision-making	NML	National Microbiology Laboratory	UK	United Kingdom
CPHLN	Canadian Public Health Laboratory Network	NRC	National Research Council Canada	VOC	Variants of concern
CPHO	Chief Public Health Officer of Canada	NSERC	Natural Sciences and Engineering Research Council of Canada	WHO	World Health Organization

## Findings

### Canada’s science advice ecosystem before the COVID-19 pandemic

#### The role of the federal government in health and science policy

The Canadian federation is composed of the Parliament of Canada and 13 subnational governments (10 provinces and three territories). Authority on matters of health is not covered by a specific provision of the 1867 *Constitution Act* and federal, provincial, and territorial (FPT) governments may all legislate on matters of health (Braën, 2004; Brideau and Brosseau, 2019; Government of Canada, 2019; Martin et al., 2018). Through the *Constitution Act*, PT governments have de jure authority to establish, maintain, and manage hospitals, asylums, and charitable institutions, while the federal government is responsible for quarantine, establishing marine hospitals, and leveraging criminal law to regulate hazardous substances (Braën, 2004; Brideau and Brosseau, 2019; Government of Canada, 2019; Martin et al., 2018). PT governments eventually became the de facto authorities for planning, organizing, and delivering health services, with the 1982 *Canada Health Act* setting out the criteria that must be met by the PT universal health insurance plans to receive federal funds (Braën, 2004; Brideau and Brosseau, 2019; Government of Canada, 2019; Martin et al., 2018). Municipal and regional governments also hold some de jure and de facto responsibilities on health matters (Bana et al., 2018; Di Ruggiero et al., 2022). Science policy in Canada does not have delineated roles and FPT governments are all involved in science matters to varied extents (Council of Canadian Academies, 2017).

#### The Federal Health Portfolio

**Fig. 1:**
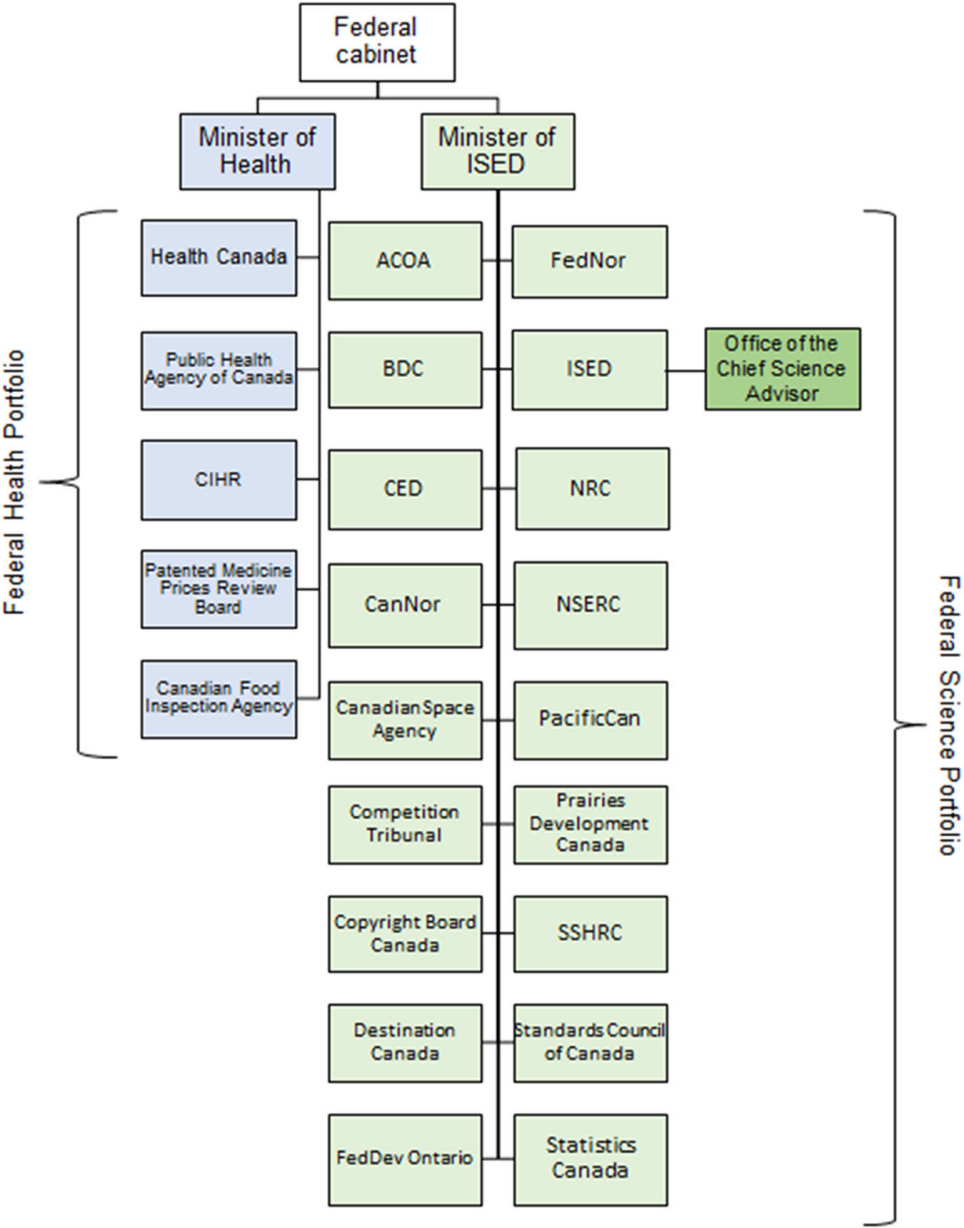
Federal Health Portfolio and Science Portfolio structure. ACOA Atlantic Canada Opportunities Agency, BDC Business Development Bank of Canada, CED Canada Economic Development for Quebec Regions, CIHR Canadian Institutes for Health Research, CanNor Canadian Northern Economic Development Agency, FedDev Ontario Federal Economic Development Agency for Southern Ontario, FedNor Federal Economic Development Agency for Northern Ontario, ISED Innovation, Science and Economic Development Canada, NRC National Research Council Canada, NSERC Natural Sciences and Engineering Research Council Canada, PacificCan Pacific Economic Development Canada, SSHRC Social Sciences and Humanities Research Council of Canada.

Canada’s Minister of Health oversees five federal departments, including Health Canada, the Canadian Institutes of Health Research (CIHR), and the Public Health Agency of Canada (PHAC)—together comprising the federal Health Portfolio (Fig. 1) (Government of Canada, 2017). Health Canada regulates the safety and efficacy of substances, including medical devices, treatments, and vaccines (Government of Canada, 2014; Marchildon et al., 2020). CIHR is composed of 13 institutes that provide health research funding (Canadian Institutes of Health Research, 2018; Marchildon et al., 2020). PHAC provides national public health leadership within the Health Portfolio and, as Canada’s national contact point for the WHO on *International Health Regulations* matters, has a prominent role in a pandemic response (Pan-Canadian Public Health Network, 2018a; Public Health Agency of Canada, 2021k).

#### Public Health Agency of Canada

In October 2003, the National Advisory Committee on SARS and Public Health, chaired by Dr. David Naylor, released the “Learning from SARS: Renewal of Public Health in Canada” report (“the Naylor Report”), recommending the creation of a federal public health agency, helmed by a Chief Public Health Officer of Canada (CPHO) (Naylor, 2003). This recommendation was the primary driver for the creation of PHAC in September 2004 (Public Health Agency of Canada et al., 2008). The *Public Health Agency of Canada Act (2006)* subsequently (i) confirmed PHAC as a legal entity responsible for governing public health functions, including emergency preparedness and response; and (ii) established the position of the CPHO—the federal government’s lead public health expert (Government of Canada, 2006, 2016; Public Health Agency of Canada et al., 2008).

The CPHO (a health professional with public health qualifications) ranks at the deputy minister^3^ level and has two primary functions: first, as a public health advisor to the Minister of Health, the President of PHAC, and the federal Cabinet^4^; and second, as a public health communicator with the public, other governments, public health authorities, and non-governmental organizations in Canada and internationally (Government of Canada, 2006). The CPHO is legally required to submit an annual report to the Minister of Health on the state of public health in Canada, which is tabled in Parliament and made public (Government of Canada, 2006). While outside the scope of the present paper, public health within each PT is governed, in part, by a similar role (the Chief Medical Officer of Health, CMOH). The responsibilities and authorities of CMOHs in advising PT governments, managing public health programs and resources, communicating with the public, and advocating on behalf of public health vary substantially across PTs (Bana et al., 2018; Eggleton et al., 2010; Fafard et al., 2018).

The PHAC departments and advisory groups that are relevant to a pandemic response are presented in Table 1. PHAC has intramural research scientists and the capacity to convene expert advisory groups to evaluate evidence for decision-making (Public Health Agency of Canada, 2021g). PHAC also funds the National Collaborating Centres (NCCs) for Public Health, which were established in 2005 and are housed within independent organizations external to PHAC to support Canada’s public health systems through evidence synthesis and knowledge translation services (National Collaborating Centres for Public Health, 2021). There are six NCCs based within universities, public health agencies, and research centers across five provinces, with each NCC working on a specific public health area of focus (National Collaborating Centres for Public Health, 2021).

**Table 1:** Public Health Agency of Canada (PHAC) programs of work.

Advisory body	Type	Mandate	Sources
Centre for Emergency Preparedness and Response (CEPR) (houses HPOC)	PHAC department	CEPR is PHAC’s central coordinating body for preventing, preparing for, responding to, and recovering from public health emergencies of both natural and human-caused origin domestically and internationally. CEPR is also responsible for developing, maintaining, and testing the Health Portfolio emergency management plans, and for serving as the Health Portfolio’s lead on counter-terrorism matters, laboratory safety, and quarantine services, with an official executive liaison post with Public Safety Canada (responsible for national security). The HPOC, housed within the CEPR, acts as the ‘focal point’ platform for emergency operations. CEPR was first created in 2000 and included in the PHAC structure after PHAC’s creation in 2004.	(Office of Audit and Evaluation, Health Canada and Public Health Agency of Canada, 2020; Public Health Agency of Canada, 2006)
Centre for Immunization and Respiratory Infectious Diseases (CIRID)	PHAC department	CIRID aims to prevent, reduce, or eliminate vaccine-preventable and infectious respiratory diseases; reduce the negative impact of emerging and re-emerging respiratory infections; and maintain public and professional confidence in immunization programs in Canada. CIRID is also tasked with implementing the National Immunization Strategy.	(Public Health Agency of Canada, 2019)
National Advisory Committee on Immunization (NACI)	Permanent external advisory committee to PHAC	NACI was established in 1964 by Health Canada as the main national body tasked with providing expert medical, scientific, and public health advice relating to vaccines and certain prophylaxis agents. Since 2004, NACI has reported to PHAC. NACI members are appointed by the Chief Public Health Officer and have expertise in pediatrics, infectious diseases, immunology, medical microbiology, internal medicine, pharmacy, nursing, and public health. NACI forms its recommendations through evidence synthesis, an assessment of the evidence of benefits and harms, and grading of the strength of the recommendations.	(Ismail et al., 2010; Public Health Agency of Canada, 2022a)
National Microbiology Laboratory (NML)	Permanent external advisory committee to PHAC	NML is Canada’s only Containment Level 4 laboratory, whose main functions include laboratory-based surveillance, emergency preparedness and response, research, and specialized services to identify diseases other labs may not be able to detect or diagnose. The Secretariat of the Canadian Public Health Laboratory Network—a network of Canada’s FPT laboratories established in 2001 to provide a forum for collaboration on issues of mutual concern—is housed at the NML.	(Canadian Public Health Laboratory Network, 2011; National Collaborating Centre for Infectious Diseases, 2021b; Public Health Agency of Canada, 2021l)
Public Health Ethics Consultative Group (PHECG)	Permanent external advisory committee to PHAC	PHECG is an external advisory group that provides advice to PHAC on ethical issues related to PHAC programs and services, as well as public health issues of national significance, to inform PHAC’s decision-making. PHECG represents the interests of the Canadian public in its advice. Between June 2020 and February 2021, PHECG released a public health ethics framework to guide COVID-19 decision-making and a guidance statement on the ethical considerations related to public reporting of COVID-19 outbreaks.	(Public Health Agency of Canada, 2021c, 2021d, 2021j)

#### Pan-Canadian Public Health Network

**Fig. 2:**
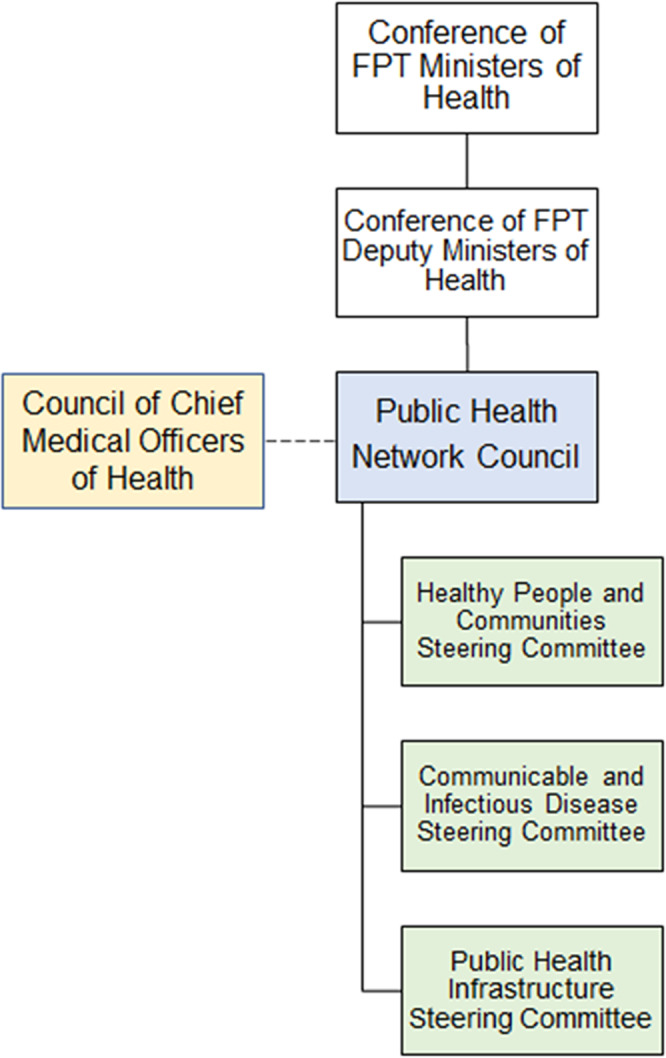
Pan-Canadian Public Health Network (PHN) structure. The 17-member PHN Council is co-chaired by the Chief Public Health Officer and a provincial or territorial Chief Medical Officer of Health and is accountable to the Conference of Deputy Ministers of Health. The Council of Chief Medical Officers of Health (a technical advisory forum composed of the Chief Public Health Officer and Chief Medical Officer of Health from provincial, territorial, and Indigenous health authorities) and three Steering Committees (each dedicated to a PHN area of focus)—(i) Healthy People and Communities, (ii) Communicable and Infectious Disease, and (iii) Public Health Infrastructure—support the PHN Council. Note: reporting relationships are depicted in solid lines, while supporting relationships are depicted in dashed lines.

Following the 2003 Naylor Report recommendations, the Pan-Canadian Public Health Network (PHN) was established in 2005 to serve as the formal intergovernmental mechanism for FPT collaboration on routine public health issues and public health threats (Pan-Canadian Public Health Network, 2016). The structure of the PHN (Fig. 2) aligns with the principle of collaborative federalism, whereby national policies are co-determined by FPT authorities as equal partners (Cameron and Simeon, 2002; Fierlbeck, 2010; Pan-Canadian Public Health Network, 2016). PHN is governed by the 17-member PHN Council, composed of senior appointed FPT civil servants that lead the health portfolios within their respective jurisdictions (such as the CPHO and CMOHs) (Pan-Canadian Public Health Network, 2020a, 2020b).

**Fig. 3:**
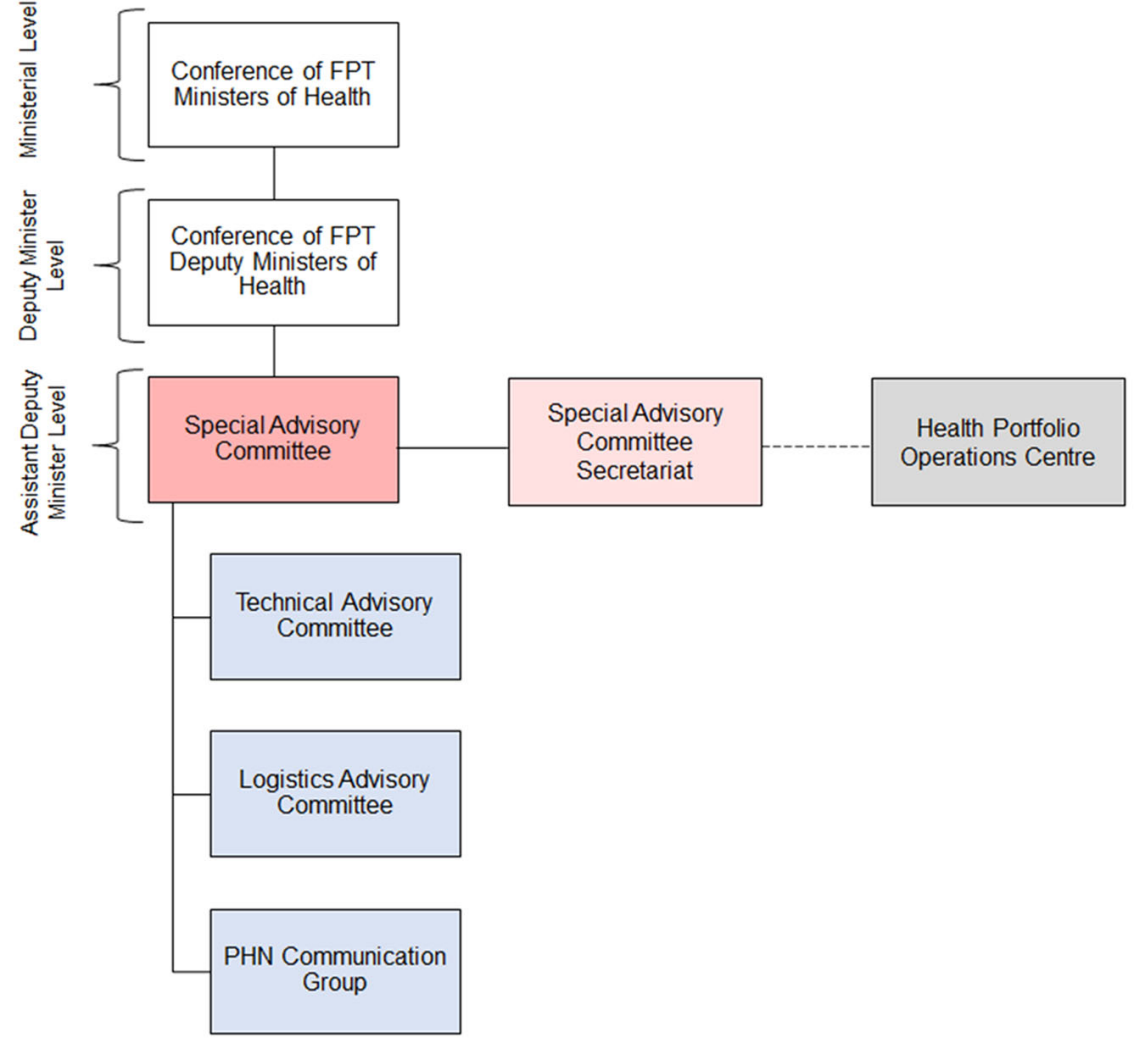
Canada’s pandemic emergency management structure. The Special Advisory Committee (SAC) may activate and deactivate three advisory groups: (i) the Technical Advisory Committee (TAC, chaired by the Communicable and Infectious Disease Steering Committee), (ii) the Logistics Advisory Committee (LAC, chaired by the Public Health Infrastructure Steering Committee), and (iii) the Pan-Canadian Public Health Network (PHN) Communication Group. Each province and territory selects representatives (typically, senior civil servants within the subnational health portfolios that have decision-making authority) to serve on the TAC and LAC. The SAC Secretariat manages the intersection between the SAC and the three response streams of the governance (the TAC, LAC, and PHN Communication Group). The TAC and LAC may convene task groups and working groups on, respectively, technical (e.g., laboratory testing, disinfection and decontamination, and case and contact surveillance) and logistical (e.g., funding allocation, engagement of external resources, use of designated sites, surge worker planning) issues and approve the protocols and guidance developed by the task groups. The PHN Communication Group coordinates public communication across the federal, provincial, and territorial authorities. Note: reporting relationships are depicted in solid lines, while supporting relationships are depicted in dashed lines.

A key function of the PHN is to activate^5^ a time-limited Special Advisory Committee (SAC) during public health emergencies to advise the Conference of FPT Deputy Ministers of Health (Fig. 3) (Pan-Canadian Public Health Network, 2018b, 2020a, 2020b). Upon activation, the SAC is composed of all members of the PHN Council and the Council of Chief Medical Officers of Health (Pan-Canadian Public Health Network, 2018b, 2020a). In its current structure (devised following the H1N1 pandemic) (Public Health Agency of Canada, 2011), the first SAC was activated in December 2016 to focus on the ongoing epidemic of opioid overdoses (Pan-Canadian Public Health Network, 2020b, 2021). A 2017 internal operational review of the PHN identified the SAC as an effective structure for managing public health emergencies (Di Ruggiero et al., 2022; Dyke, 2017).

#### The Federal Science Portfolio

The Minister of Innovation, Science and Economic Development (ISED) oversees the Science Portfolio, which is composed of 18 federal departments and agencies (Fig. 1), including ISED, National Research Council Canada (NRC), Natural Sciences and Engineering Research Council of Canada (NSERC), Social Sciences and Humanities Research Council of Canada (SSHRC), and Statistics Canada (Innovation, Science and Economic Development Canada, 2021f). ISED leads the Science Portfolio and is mandated to support business innovation and scientific research (Innovation, Science and Economic Development Canada, 2018). The NRC is Canada’s largest federal research and development organization, responsible for bringing research innovations to market, as legislated in the *National Research Council Act (1985)* (Government of Canada, 1985a; National Research Council of Canada, 2019). NSERC and SSHRC are funding agencies for natural sciences and technology and humanities and social sciences, respectively. Together, CIHR (in the Health Portfolio), NSERC, and SSHRC are referred to as the Tri-Agencies or the Government’s granting councils, which are coordinated by the Canada Research Coordinating Committee, created in 2018 (Canada Research Coordinating Committee, 2020). Statistics Canada is Canada’s central statistical office that is governed by the *Statistics Act (1985)* and is responsible for conducting the Census and over 350 other surveys on the health, social, and economic activities of the public (Government of Canada, 1985b; Statistics Canada, 2020).

Each Science Portfolio department and agency may conduct stakeholder consultations and public opinion research, and convene expert advisory groups (Innovation, Science and Economic Development Canada, 2020). Several arm’s-length research organizations are funded by the Science Portfolio, such as Genome Canada (a non-profit organization focused on genomics-based technologies) (Genome Canada, 2021a) and the Council of Canadian Academies (CCA) (a non-profit organization focused on synthesis and expert appraisal of the best available evidence on public policy matters) (Council of Canadian Academies, 2021; Quirion et al., 2016).

#### Office of the Chief Science Advisor

Prior to 2018, Canada did not have a formalized apparatus for providing science advice to inform the federal government’s decision-making (Office of the Chief Science Advisor, 2020a). For a comprehensive account of the history of science advisory systems in Canada, we direct interested readers to the work by Quirion et al. (2016). Briefly, between 2003 and 2008, the Office of the National Science Advisor was instituted to, first, advise the Prime Minister of Canada on broad policy issues (Quirion et al., 2016), and then, to advise the Minister of Industry on science and technology research and development policy (Industry Canada, 2008; Quirion et al., 2016). The Office of the National Science Advisor also supported the Royal Society of Canada, the Canadian Academy of Engineering, and the Canadian Academy of Health Sciences in founding the CCA in 2005, which is seen as one of the Office’s major accomplishments (CBC News, 2008; Quirion et al., 2016).

Following a change in the national governing party, the Office of the National Science Advisor was terminated in March 2008 and replaced with the Science, Technology and Innovation Council (STIC)—an advisory committee within the Department of Industry (CBC News, 2008; Quirion et al., 2016). Considering the narrow remit of the STIC, limited to technological innovation and development (Royal Society of Canada, 2015), many scientists and the public viewed the closure of the Office of the National Science Advisor as a dissolution of a necessary ‘voice at the table’ for science within the government and a crucial line of communication between Canada’s scientific community and the federal government (CBC News, 2008; Quirion et al., 2016). The STIC was wound down in 2018 following the recommendations of the 2017 Fundamental Science Review (an independent review of the federal science and research ecosystem) (Canada’s Fundamental Science Review and Naylor, 2017; Evidence for Democracy, 2019).

The Office of the Chief Science Advisor (OCSA) (currently in operation) was established by the Liberal government in 2018 within the ISED department, reporting to the Minister of ISED and the Prime Minister (Office of the Chief Science Advisor, 2020a). The mandate of the Chief Science Advisor is (i) to develop and implement guidelines to ensure that government scientists are able to speak freely about their work and that government science is available to the public; (ii) to ensure that scientific analyses are considered in the federal government’s decisions; (iii) to recommend ways for improving the science advisory function within the federal government; and (iv) to recommend ways for the federal government to better support quality scientific research (Office of the Chief Science Advisor, 2018). In addition, the Chief Science Advisor delivers an annual report, which is tabled in Parliament^6^ and made public, to the Prime Minister and to the Minister of ISED on OCSA activities; coordinates expert advice to the federal Cabinet and the Minister of ISED; and supports the dialogue between scientists within and outside of government in Canada and internationally (Office of the Chief Science Advisor, 2018).

In its first year, the OCSA established the Departmental Science Advisors (DSA) Network—a network of lead science officials and subject matter experts that work closely with senior officials within each federal department and support the mandate of the Chief Science Advisor (Office of the Chief Science Advisor, 2019, 2020a, 2021b). The DSA Network, chaired by the Chief Science Advisor, aims to coordinate science advice across the federal portfolios (including the Health Portfolio), and, as of 2021, has eight members from different federal departments, including PHAC, Health Canada, and the NRC (Office of the Chief Science Advisor, 2021b). The DSA Network meets on a monthly basis to collaborate and serve as peer-reviewers on multi-departmental government science initiatives (Office of the Chief Science Advisor, 2021b).

#### Federal guidelines for science advice in a pandemic emergency

Emergency management in Canada falls within the National Security Portfolio, which is managed by the Minister of Public Safety. Canada’s first National Security Policy (“Securing an Open Society: Canada’s National Security Policy”) was released after SARS in 2004 and served to position pandemics among the key national security priorities (alongside intelligence, emergency planning and management, and transport, border, and international security) (Canada and Privy Council Office, 2004). The first National Security Policy also supported the implementation of PHAC and the Office of the CPHO (Canada and Privy Council Office, 2004). The federal government’s “all-hazards” emergency management approach is guided by the Federal Emergency Response Plan (2011) (Public Safety Canada, 2011), with the legal and policy scaffolding provided by, respectively, the *Emergency Management* Act **(**2007) (Government of Canada, 2007) and the Federal Policy on Emergency Management (2009) (Public Safety Canada, 2009). In a national emergency, one of 13 federal departments is designated as the “primary institution” that convenes and coordinates national stakeholders in emergency response (Public Safety Canada, 2011). The Health Portfolio is the primary federal institution for all public health emergencies, including pandemics (Pan-Canadian Public Health Network, 2018a; Public Safety Canada, 2011). The Health Portfolio Operations Centre (HPOC) within the Centre for Emergency Preparedness and Response (first created in 2000 and moved to PHAC in 2004) (Canada and Privy Council Office, 2004; Public Health Agency of Canada, 2006) acts as the focal point for coordinating emergency management across federal departments, PT authorities, and other actors during public health events (Pan-Canadian Public Health Network, 2018b, 2018a). The HPOC supports the PHN SAC and its committees via the SAC Secretariat (Pan-Canadian Public Health Network, 2018b).

Under the *Emergency Management Act*, each federal Minister is responsible for developing, testing, and maintaining an emergency plan specific to their portfolio (Pan-Canadian Public Health Network, 2018a). Two such documents outline the roles and responsibilities of the Health Portfolio in a pandemic emergency response: the FPT Public Health Response Plan for Biological Events (2018) and the Canadian Pandemic Influenza Preparedness: Planning Guidance for the Health Sector (CPIP) (2018). In alignment with the principles outlined in the “all-hazards” Federal Emergency Response Plan, the FPT Public Health Response Plan for Biological Events provides the overarching FPT governance framework for responding to public health events, including the activation of the Health Portfolio emergency management structure (Pan-Canadian Public Health Network, 2018b, 2018a). The CPIP provides complementary pandemic-specific guidance, viewed as applicable to both influenza and other respiratory viruses (Pan-Canadian Public Health Network, 2018a, 2018b; Public Health Agency of Canada, 2021f). The CPIP was first published after the SARS outbreak in 2004, majorly revised after the 2009 H1N1 pandemic, and is reviewed every five years (Pan-Canadian Public Health Network, 2018a). PTs also have their own emergency management and pandemic response plans that function in concert with the federal plans.

Evidence-informed decision-making is cited as a core principle in Canada’s national pandemic response plans (Pan-Canadian Public Health Network, 2018a, 2018b). The CPIP outlines four components of a pandemic knowledge generation and dissemination strategy: rapidly identifying research needs in specific areas of the response; leveraging existing collaborations to mount rapid primary research; performing evidence syntheses for decision-making; and forming collaborations between PHAC, Health Canada, and other FPT, academic, and public health institutions to learn from the pandemic in the post-pandemic period (Pan-Canadian Public Health Network, 2018a). Notably, the CPIP does not provide specific guidance on the procurement of expertise or the role of the Science Portfolio.

### Canada’s science advice ecosystem during the COVID-19 pandemic

#### Mobilization of pre-established science advisory bodies

At the outset of the COVID-19 pandemic, the federal response was coordinated by the Incident Response Group—a time-limited Cabinet group of federal ministers chaired by the clerk of the Privy Council Office/Secretary of the Cabinet that is convened by the Prime Minister in national crises, such as domestic and international terrorism threats (Cappe, 2020; Prime Minister of Canada Office, 2021b). A pandemic-specific federal decision-making group—the Sub-Committee on the Federal Response to COVID-19^7^, mandated to provide “whole-of-government leadership, coordination, and preparedness for a response to, and recovery from, COVID-19”—was announced on March 4, 2020 by the Prime Minister (Cappe, 2020; Prime Minister of Canada Office, 2021c).

The Health Portfolio emergency management structure for the COVID-19 pandemic was established in January 2020 and followed the existing pandemic playbook. The HPOC and the FPT Public Health Response Plan for Biological Events were triggered mid-January 2020 (Office of Audit and Evaluation, Health Canada and Public Health Agency of Canada, 2020). Upon official confirmation of the first COVID-19 case in Canada by the National Microbiology Laboratory (NML), the Council of Chief Medical Officers of Health and the FPT SAC on COVID-19, along with its committees and their supporting task forces and working groups (e.g., Surveillance Expert Working Group, Canadian Pandemic Influenza Plan Task Group, and the Public Health Working Group on Remote and Isolated Communities^8^), were activated on January 28, 2020 (Office of Audit and Evaluation, Health Canada and Public Health Agency of Canada, 2020; Pan-Canadian Public Health Network, 2022; Public Health Agency of Canada, 2021f). Over 14 public statements had been issued by the Council of Chief Medical Officers of Health on behalf of the SAC by December 2021, with 10 statements focused on COVID-19 vaccination, endorsing the National Advisory Committee on Immunization (NACI) COVID-19 vaccination guidance (Pan-Canadian Public Health Network, 2022; Public Health Agency of Canada, 2022a). Since the start of the pandemic, the NML has provided leadership in developing COVID-19 testing assays, scaling up testing capacity across the country and sharing epidemiological and surveillance information through the Canadian Public Health Laboratory Network (CPHLN), and supporting research on medical countermeasures and the impact of emerging variants of concern (VOC) (Public Health Agency of Canada, 2021f, 2022c).

New collaborations between the Health Portfolio and other federal departments emerged during the COVID-19 pandemic. For example, in March 2020, PHAC sought the expertise of behavioral scientists at the Impact and Innovation Unit (situated within the Privy Council Office since 2017) to inform PHAC’s pandemic messaging to promote public uptake of public health protective measures (Impact and Innovation Unit, 2018, 2020; Impact Canada Initiative, 2022b). The Impact and Innovation Unit was explicitly acknowledged by the CPHO for the first time in February 2021 in the context of the federal government’s efforts to promote vaccine confidence (Delacourt, 2021); however, the CPHO’s statements generally aligned with the Impact and Innovation Unit’s public messaging campaigns since the first wave of the pandemic (Impact Canada Initiative, 2022c, 2022a; McCoy et al., 2020). Another notable example is the Wastewater Surveillance Program for COVID-19, comprised of scientists at the NML and Statistics Canada (National Collaborating Centre for Infectious Diseases, 2021a; Public Health Agency of Canada, 2021h, 2021i). Wastewater surveillance has supported targeted outbreak prevention and detection of circulating VOCs in several communities in Canada—particularly where widespread testing may not have been available (National Collaborating Centre for Infectious Diseases, 2021a; Public Health Agency of Canada, 2021h, 2021i). As of December 2021, PHAC was leading four working groups on VOCs in wastewater, wastewater laboratory detection methods, and data modeling and epidemiological interpretation (National Collaborating Centre for Infectious Diseases, 2021a).

**Fig. 4:**
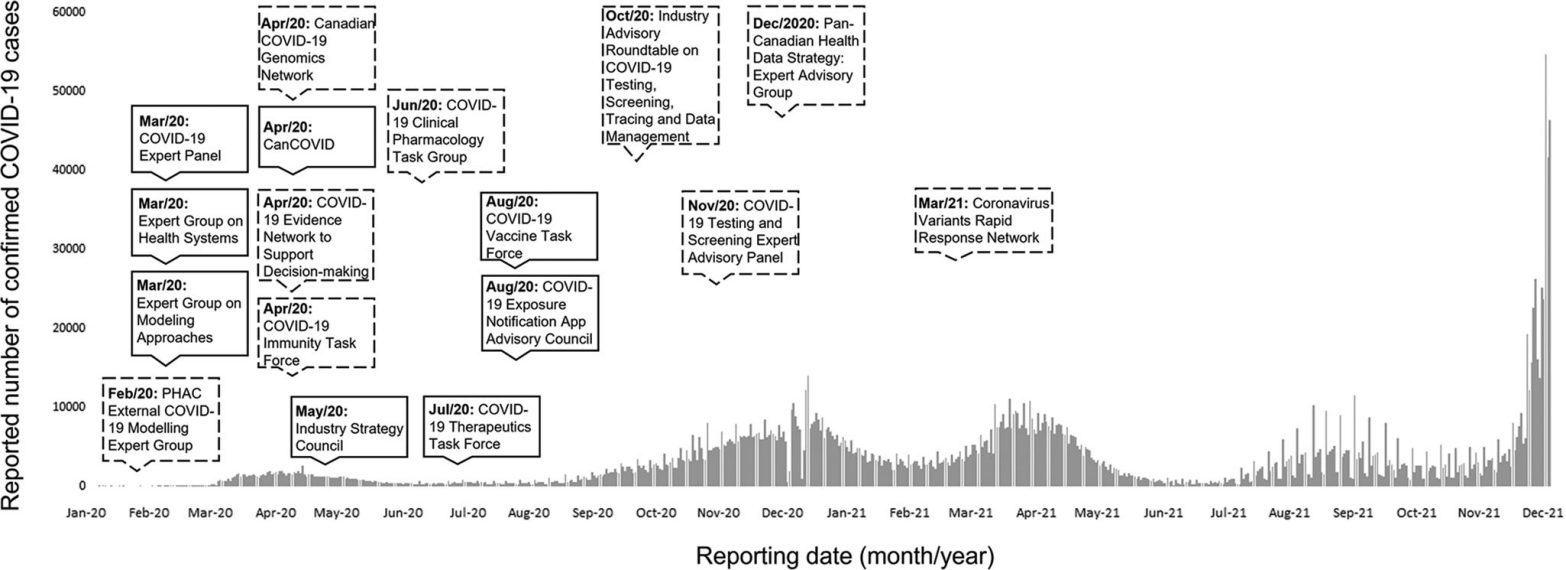
Timeline of new federal science advisory bodies formed in the first two years of the COVID-19 pandemic. Note: dashed borders represent bodies established by the Health Portfolio; solid borders represent bodies established by the Science Portfolio. COVID-19 case data (reporting dates: January 25, 2020-December 31, 2021) was retrieved from the COVID-19 Canada Open Data Working Group: https://opencovid.ca/work/dataset/.

The interim FPT Public Health Response Plan for Ongoing Management of COVID-19 was released in August 2020 (Public Health Agency of Canada, 2020a) and revised in April 2021 (Public Health Agency of Canada, 2021f) to outline the common near-, mid-, and long-term goals for COVID-19 management in Canada in the context of the overall pandemic response objective^9^ and a “reasonable worst-case scenario”. This interim plan “draws extensively” from the 2018 FPT Public Health Response Plan for Biological Events and the CPIP to provide guidance for the federal COVID-19 response as a living document, updated with evolving scientific knowledge until COVID-19 activity in Canada has reached a “low, manageable, and tolerable level” (Public Health Agency of Canada, 2021f). The August 2020 version of the interim plan described the federal government’s prioritization of investigations related to COVID-19 mathematical modeling, social and behavioral sciences, serological and wastewater surveillance, and genomic innovation (Public Health Agency of Canada, 2020a); this was also reiterated in the April 2021 updated version of the plan (Public Health Agency of Canada, 2021f). As detailed next, these priorities were pursued by establishing new science advisory bodies between the spring and fall of 2020 (i.e., the first two waves of the COVID-19 pandemic in Canada) (Fig. 4) to (i) convene relevant experts, and (ii) support real-time primary data collection and rapid evidence syntheses.

#### Procurement of expertise through new science advisory bodies

##### Expert advisory groups established by the Health Portfolio

The time-limited expert advisory groups convened by the Health and Science Portfolios during the COVID-19 pandemic are detailed in Table 2. The PHAC External COVID-19 Modeling Expert Group was created in February 2020 to support PHAC’s internal modeling group (formed in January 2020 and largely composed of modelers and epidemiologists at the NML) (National Collaborating Centre for Infectious Diseases, 2022; Public Health Agency of Canada, 2022c) in developing epidemiological models to estimate the infection burden, impact of public health measures, and factors contributing to chains of transmission (Public Health Agency of Canada, 2021f, 2022b). The Pan-Canadian Health Data Strategy Expert Advisory Group, convened by PHAC in December 2020, was mandated to provide recommendations to the Conference of Deputy Ministers of Health on the creation, collection, storage, and use of health data across FPTs during and beyond public health emergencies like COVID-19 (Public Health Agency of Canada, 2021m). The Group was expected to conclude its mandate by winter 2022, and by December 2021, the Group had held 10 monthly meetings and released two public reports identifying challenges and opportunities for modernizing Canada’s health data ecosystem (Public Health Agency of Canada, 2021e, 2022d). The Ad-hoc COVID-19 Clinical Pharmacology Task Group was convened in June 2020 to advise PHAC’s Director General of the Centre for Immunization and Respiratory Infectious Diseases on pharmaceutical products for treatment and chemoprophylaxis of COVID-19 (Government of Canada, 2020c, 2020d). Owing to the entry of COVID-19 vaccines in Canada, the Task Group concluded its mandate on March 30, 2021, and its recommendation statements are no longer publicly accessible (Government of Canada, 2020c).

**Table 2:** Time-limited expert advisory bodies convened by the Health and Science Portfolios to procure expertize during the COVID-19 pandemic.

Advisory body (dates active)	Mandate	Portfolio	Federal department (reporting to)	Members	Public outputs	Sources
**General COVID-19 strategy**						
Industry Strategy Council (May 2020-)	To advise on (i) the scope and depth of the COVID-19 pandemic’s impact on industries and inform the federal government’s understanding of specific sectoral pressures; and (ii) to serve as a means to coordinate business communities’ input on the impact of COVID-19.	Science	ISED (Minister of ISED)	1 chair and 9 external members from the private sector who are chairs of ISED’s Economic Strategy Tables (including Advanced Manufacturing, Agri-Food, Clean Technologies, Digital Industries, Health/Biosciences, Resources of the Future, Retail, Tourism and Hospitality, and Transportation). 2 ex-officio members: the Chief Science Advisor and the Deputy Minister of ISED.	Between June 2020 and September 2020, the Council had held 11 meetings and released 1 public report on economic recovery from COVID-19, with recommendations spanning income assistance and employment programs, government investments into the hardest-hit sectors and biomedical manufacturing, and opportunities for public-private partnerships.	(Innovation, Science and Economic Development Canada, 2021a)
Pan-Canadian Health Data Strategy Expert Advisory Group (December 2020-)	To advise on (i) strategic direction for the use of health system, population, and public health data to improve the health of Canada and Canadians; (ii) principles to guide the creation, collection, storage, and use of data; and (iii) a practical and phased roadmap for the implementation of measures to sustainably address areas of greatest opportunity and impact.	Health	PHAC (Conference of Deputy Ministers of Health)	1 chair and 19 external members from PT health agencies, academia, healthcare research centers, and non-profit organizations, with expertise in health system, public health, and population health data with perspectives on analytics, data management, and privacy.	Between December 2020 and December 2021, the Group had held 10 monthly meetings and released 2 reports (June and November 2021) identifying a vision for Canada’s health data ecosystem, identifying the root causes that have prevented progress, highlighting opportunities for improvement, and developing practical recommendations.	(Public Health Agency of Canada, 2021e, 2021m, 2022d)
COVID-19 Expert Panel (March 2020-)	To advise the Chief Science Advisor on the latest scientific developments relevant to COVID-19. This information assists the Chief Science Advisor in providing current and cross-disciplinary advice to the Prime Minister and other federal departments.	Science	OCSA (Chief Science Advisor)	19 internal and external members from PHAC, Health Canada, academia, and healthcare research centers, with expertise in disease modeling, risk and behavioral sciences, and biomedical and clinical sciences.	Published 5 reports on COVID-19 and children (July 2020), COVID-19 and long-term care (April-May 2020, 2 parts), the role of bioaerosols and indoor ventilation in COVID-19 transmission (September 2020), scientific considerations for COVID-19 vaccine certificates (March 2021), and COVID-19 vaccine-associated myocarditis/pericarditis (July 2021). The following task forces, assembled to support the Expert Panel, have not released public reports at the time of writing: reprocessing of respirators/N95 masks, ventilators, virtual care, data analytics, optimal use of health system capacity, and long-term care systemic issues.	(Office of the Chief Science Advisor, 2020a, 2021a, 2022)
**Health system planning**						
Expert Group on Health Systems (March 2020-)	To obtain practical opinion from domain experts in order to inform the Chief Science Advisor’s advice to the federal government regarding health services needs and innovation. This group is a sub-committee of the OCSA COVID-19 Expert Panel.	Science	OCSA (Chief Science Advisor)	2 co-chairs (internal): Deputy Minister of Health and the Chief Science Advisor. 11 external members from the academic sector.	No public outputs specific to this group.	(Office of the Chief Science Advisor, 2020a, 2020b)
**COVID-19 mathematical modeling**						
Expert Group on Modeling Approaches (March 2020-)	To review modeling approaches to predict and manage disease spread, identify hot spots in PTs, and inform recovery strategies, data accessibility, and data gaps. This group is a sub-committee of the OCSA COVID-19 Expert Panel.	Science	OCSA (Chief Science Advisor)	2 co-chairs (internal): Deputy Minister of Health and the Chief Science Advisor. 10 internal and external members from PHAC and the academic sector.	No public outputs specific to this group.	(Office of the Chief Science Advisor, 2020a, 2020c)
External COVID-19 Modeling Expert Group (February 2020-)	To develop epidemiological models to estimate the numbers of cases, hospitalizations, and deaths; evaluate the effects of public health measures at the national and community levels; and assess the factors linked to chains of transmission, including new VOCs.	Health	PHAC (Not specified)	33 external members from academia and 43 individuals from FPT governmental and public health organizations (as of April 2021).	First modeling technical briefing released in April 2020; 22 monthly modeling updates published by December 2021.	(Public Health Agency of Canada, 2020a, 2022b)
**COVID-19 clinical therapeutics**						
Ad-hoc COVID-19 Clinical Pharmacology Task Group (June 2020-March 2021)	To provide medical and scientific advice on an ad-hoc basis relating to pharmaceutical products for treatment and chemoprophylaxis of COVID-19 in humans. PHAC retains all decision-making authority, and policy-making and investigatory responsibilities.	Health	PHAC (Director General of the CIRID)	2 co-chairs: 1 internal from PHAC and 1 external from the academic sector. 6 external members from the academic sector, with expertise in infectious disease, microbiology, pharmacology, and pharmaceutical sciences, recruited from membership lists of relevant professional organizations.	Provided recommendation statements on remdesivir, hydroxychloroquine, dexamethasone, and monoclonal antibodies, which were selected for risk-benefit appraisal because they had “sufficient” evaluative evidence (i.e., randomized controlled trials that were adequately powered to provide evidence of efficacy and safety). Recommendation statements are no longer publicly available.	(Government of Canada, 2020c, 2020d)
COVID-19 Therapeutics Task Force (July 2020-February 2021)	To provide expert advice on COVID-19 therapeutics by assessing and prioritizing COVID-19 therapeutics projects seeking federal government support in Canada.	Science	NRC (Minister of ISED)	Core group of 8 external members from life sciences industry and 11 scientific advisors from academia and healthcare research centers with expertise in infectious disease, immunology, virology, and microbiology.	No outputs have been made public. According to a January 2021 public presentation on the Task Force’s progress, the Task Force provided 11 advice letters to the federal Ministers and assessed over 40 Strategic Innovation Fund proposals and over 40 international COVID-19 therapeutics candidates.	(CanCOVID, 2021b; Innovation, Science and Economic Development Canada, 2021b)
**COVID-19 vaccination**						
COVID-19 Vaccine Task Force (August 2020-)	To prioritize COVID-19 vaccine projects seeking support in Canada; attract and partner with developers of promising non‑Canadian COVID-19 vaccine candidates; optimize the tools needed to develop COVID-19 vaccines; support research and development, and supply chain coordination for COVID-19 vaccine projects; and facilitate solutions to manufacture COVID-19 vaccines in Canada.	Science	NRC (Minister of ISED)	2 co-chairs (external): 1 from academic sector and 1 from the biotechnology sector. 11 external members from the academic and biotechnology sectors, with expertise in biochemistry, microbiology, immunology, and infectious diseases. 4 ex-officio members, including the Chief Science Advisor, the PHAC President, and the Deputy Ministers of Health and ISED.	No outputs have been made public. In August 2020, the Minister of Public Services and Procurement announced that following the Task Force’s recommendations, the Government of Canada has entered into two agreements with Pfizer and Moderna to secure their mRNA vaccine candidates, pending Health Canada regulatory approval. The Minister of ISED further announced an investment of up to $56 million to support COVID-19 vaccine trials in Canada.	(CanCOVID, 2021b; Government of Canada, 2020a, 2020b)
**COVID-19 screening, testing, and tracing**						
COVID-19 Testing and Screening Expert Advisory Panel (November 2020-)	To advise the Minister of Health on science and policy related to COVID-19 testing and screening approaches (including emerging testing technologies and their combinations, different applications of testing, and testing strategies for specific settings).	Health	Health Canada (Minister of Health)	12 external members from governmental, academic, and non-governmental public health and healthcare organizations with expertise in epidemiology, virology, data analytics, pediatrics, healthcare provision, and technology assessment. 8 ex-officio members, including Chief Science Advisor, the Deputy CPHO, the Deputy Ministers of Health and ISED, and the PHAC and NRC Presidents.	Between November 2020 and April 2021, the Panel had held 24 meetings and published 5 reports with recommendations on optimizing testing and screening for COVID-19 in Canada; COVID-19 self-testing approaches; testing and screening in long-term care and school settings; as well as at Canada’s borders.	(Government of Canada, 2021; Health Canada, 2021a, 2021b; Public Health Agency of Canada, 2021a, 2021n)
Industry Advisory Roundtable on COVID-19 Testing, Screening, Tracing and Data Management (October 2020-)	To advise on: (i) the role of COVID-19 testing, screening, tracing, and data to address challenges faced by Canada’s industrial sectors and to assist in identifying solutions; (ii) development of industry workplace pilots to determine best approaches for COVID-19 testing and screening in the workplace; and (iii) guidance on rolling out testing more broadly if pilots are successful.	Health	Health Canada (Minister of Health)	16 external members from academia and the private sector (including transportation, tourism, manufacturing, retail, life sciences, energy, organized labor, construction, and telecommunications).	Published 4 reports with recommendations on workplace COVID-19 testing and screening programs, as well as the need to balance COVID-19 testing with free flow of people and goods across Canada’s borders.	(Health Canada, 2021c)
COVID-19 Exposure Notification App Advisory Council (August 2020-)	To ensure that the nation-wide COVID Alert exposure notification smartphone application (“app”) (developed with Health Canada’s support) meets the highest standards in public health outcomes, technology, and privacy.	Science	ISED (Deputy Ministers of ISED, Health, and Intergovernmental Affairs)	2 co-chairs (external) with expertise in entrepreneurship, software development, and privacy law. 9 members from the academic, public, and private sector, with expertise in cybersecurity, privacy and surveillance, computer science, and public health.	Between August 2020 and May 2021, the Council had held 15 meetings and published 1 interim report (February 2021) on the social and economic determinants of COVID Alert app adoption, retention, and use.	(Innovation, Science and Economic Development Canada, 2021e)

The information presented in this table reflects the status of the expert advisory groups at the time of data collection (December 2021). By “internal” members, we mean individuals employed by the Government of Canada departments and agencies. All advisory group members were based within Canada. Mentions of “academia” or the “academic sector” generally denote individuals employed within Canadian universities.

*CIRID* Centre for Immunization and Respiratory Infectious Disease, *CPHO* Chief Public Health Officer, *FPT* federal, provincial, territorial, *ISED* Innovation Science and Economic Development, *NRC* National Research Council Canada, *OCSA* Office of the Chief Science Advisor, *PHAC* Public Health Agency of Canada, *PT* provinces and territories, *VOC* variants of concern.

Health Canada established the COVID-19 Testing and Screening Expert Advisory Panel in November 2020 to advise the Minister of Health on science and policy issues related to COVID-19 testing and screening, including strategies for different testing technologies, populations, and settings (Health Canada, 2021a; Public Health Agency of Canada, 2021a, 2021n). Between November 2020 and April 2021, the Panel had held 24 meetings and published five reports (Government of Canada, 2021). At the time of writing, the Panel has not been disbanded; however, it has also not been asked to provide advice since the publication of its fifth report in August 2021. The Industry Advisory Roundtable on COVID-19 Testing, Screening, Tracing and Data Management was launched in October 2020 to complement the technical work of the COVID-19 Testing and Screening Expert Advisory Panel and advise on implementing COVID-19 testing in industry workplace settings (Health Canada, 2021c). The Roundtable was formed by Health Canada in consultation with ISED’s Industry Strategy Council, created in May 2020 “to coordinate business communities’ input on the impact of COVID-19” (Innovation, Science and Economic Development Canada, 2021a).

##### Expert advisory groups established by the Science Portfolio

In March 2020, the OCSA established the COVID-19 Expert Panel, composed of 19 experts internal and external to the federal government, to support the Chief Science Advisor in developing evidence-informed advice for federal decision-makers (Office of the Chief Science Advisor, 2020a, 2021a). Subgroups of the Expert Panel—the Expert Group on Health Systems and the Expert Group on Modeling Approaches—were formed in mid-to-end of March 2020 (Office of the Chief Science Advisor, 2020a, 2020b, 2020c). The Expert Group on Health Systems was assembled to procure information on health service needs and system planning at the request of Health Canada (Office of the Chief Science Advisor, 2020a, 2020b). The objective of the Expert Group on Modeling Approaches is to review mathematical modeling methodologies to predict COVID-19 spread and identify hot spots, recovery strategies, and data gaps (Office of the Chief Science Advisor, 2020a, 2020c). As the pandemic evolved, the Chief Science Advisor drew on the Expert Panel and the two Expert Groups to develop public reports on scientific standards and best practices on emerging issues, including respirators, ventilators, virtual care, long-term care, data analytics, optimal use of health system capacity, COVID-19 in children, vaccine-associated cardiovascular complications, vaccination certificates, and the role of bioaerosols and indoor ventilation in COVID-19 transmission (Office of the Chief Science Advisor, 2020a, 2022).

Three expert advisory groups were convened by other Science Portfolio departments. In July 2020, the Government of Canada began rolling out the COVID-19 exposure notification smartphone application (“app”) developed through Health Canada—COVID Alert (Bhatia et al., 2020). The COVID-19 Exposure Notification App Advisory Council, established by ISED to “ensure that the app meets the highest standards in public health outcomes, technology, and privacy”, had its first meeting on August 5, 2020 (Innovation, Science and Economic Development Canada, 2021e). By May 26, 2021, the Council had held 15 meetings and released its first interim report on the social and economic determinants of COVID Alert adoption, retention, and use in February 2021 (Innovation, Science and Economic Development Canada, 2021e). The COVID-19 Therapeutics Task Force was formed by the NRC in July 2020 to advise the Minister of ISED on the procurement, prioritization, and manufacturing of COVID-19 therapeutic products seeking to enter the Canadian market, “until [it is possible to] immunize Canadians on a national scale with an effective vaccine” (Innovation, Science and Economic Development Canada, 2021b). The Therapeutics Task Force concluded its mandate on February 28, 2021 (Innovation, Science and Economic Development Canada, 2021b). The COVID-19 Vaccine Task Force, convened to advise the Minister of ISED on the prioritization, development, manufacturing, and supply chain coordination of domestic and international vaccine projects, was first publicly acknowledged by the NRC in August 2020 (Government of Canada, 2020b). No outputs of the Therapeutics and Vaccine Task Forces have been made public; however, the Ministers of ISED and Public Services and Procurement cited the recommendations of both Task Forces in their August 2020 announcements regarding new contracts with vaccine candidate suppliers and investments in domestic clinical trials of COVID-19 vaccines and therapeutics (Government of Canada, 2020a).

#### Procurement of evidence through new science advisory bodies

##### Primary data collection

The federal government funded three major research partnerships during the COVID-19 pandemic to scale up primary data collection to inform decision-making at FPT levels. In February 2021, the Government of Canada announced the VOC Strategy, which included an investment of $53 million to support and scale up VOC surveillance and sequencing efforts (Public Health Agency of Canada, 2021b). The Canadian COVID-19 Genomics Network (CanCOGeN)—a partnership between PHAC, Health Canada, CPHLN, CIHR, healthcare facilities, and academic researchers, established by Genome Canada in April 2020—was funded through the VOC Strategy to (i) provide pan-Canadian coordinated cross-agency viral and human host sequencing to track viral origin, spread, and evolution, to inform time-sensitive decision-making relevant to Canada’s health authorities; and (ii) contribute to building national data infrastructure to address future infectious disease outbreaks and emergencies (Genome Canada, 2021b; Genome Canada Canadian COVID-19 Genomics Network and Canadian Public Health Laboratory Network CanCOGeN Working Group, 2021; Public Health Agency of Canada, 2021k). At the time of writing, CanCOGen was leading two viral sequencing projects to understand COVID-19 spread and host responses—VirusSeq (sequencing of viral samples from people testing positive for COVID-19) and HostSeq (sequencing of genomes of people diagnosed with COVID-19) (Genome Canada, 2021c; Public Health Agency of Canada, 2021b).

The Coronavirus Variants Rapid Response Network (CoVaRR-Net)—a network of interdisciplinary researchers chaired by the Deputy Minister of Health—was also funded through the federal VOC Strategy in March 2021 to develop mechanisms (e.g., a national biobank, wastewater monitoring, and resource-sharing agreements) to facilitate rapid research on VOCs from the immunology, laboratory science, genomics and sequencing, modeling and computational biology, and public health and social systems perspectives (CanCOVID, 2021c; CoVaRR-Net, 2021). CoVaRR-Net collaborates with the NML at PHAC, CanCOGeN, and PT public health labs, with plans to also establish an Indigenous Network for VOCs (CoVaRR-Net, 2021). In the long-term, CoVaRR-Net intends to morph into a Pandemic Preparedness Network with established channels to rapidly mobilize findings to Canadian and international decision-makers (CanCOVID, 2021c; CoVaRR-Net, 2021).

Lastly, in April 2020, the Health Portfolio funded the COVID-19 Immunity Task Force (CITF) to support, fund, and harmonize research on COVID-19 seroprevalence and vaccine effectiveness, safety, and immunogenicity, to develop population immunity models that can guide FPT pandemic decisions (COVID-19 Immunity Task Force, 2021a). To conduct its work, the CITF has developed partnerships with FPT governments, public health agencies, academic institutions, and community organizations, and the CITF Secretariat is housed at McGill University in Canada (COVID-19 Immunity Task Force, 2021a). In its first year, the CITF funded the SeroTracker—a seroprevalence dashboard using Canadian and international serosurvey data (COVID-19 Immunity Task Force, 2021b). Studies on vaccine safety and effectiveness are overseen by the Vaccine Surveillance Reference Group—an expert group reporting to the President of PHAC, NACI, and other FPT actors that is independent from the CITF, but is supported by its Secretariat (COVID-19 Immunity Task Force, 2021c).

##### Evidence syntheses

The mounting demand for evidence reviews across federal departments and PT authorities, caused by the rapid pace and low methodological quality of much of the early COVID-19 research, exceeded the capacity of PHAC departments (e.g., the Emerging Science Group) to perform this work internally. Arm’s-length actors, such as the NCCs and the CCA, were leveraged to build surge capacity for evidence review, synthesis, and dissemination, as these are the core functions of these organizations. However, while these channels were well-equipped for long-term projects, there remained a need for rapid short-term evidence reviews on focused research questions.

Two initiatives were developed to meet this demand: CanCOVID and the COVID-19 Evidence Network to Support Decision-making (COVID-END) (Public Health Agency of Canada, 2021k). CanCOVID—an invitation-based network of verified members of the COVID-19 research and response community (both within Canada and internationally)—was created by the OCSA in April 2020 with ISED funding, following the recommendations of the Expert Panel on COVID-19, the DSA Network, and the U15 Group of Canadian Research Universities (a collective of Canada’s most research-intensive universities) (CanCOVID, 2021a; Office of the Chief Science Advisor, 2020a; Public Health Agency of Canada, 2021k). The mission of CanCOVID is to foster collaborations and rapid knowledge sharing within the scientific community, and to provide a line of communication between scientists and decision-makers (CanCOVID, 2021a). The CanCOVID platform consists of moderated subject-based channels hosting rapid evidence reviews and other resources on over 20 topics, such as COVID-19 in schools, testing and tracing, mental health and wellbeing, and health equity (CanCOVID, 2021a; Office of the Chief Science Advisor, 2020a).

COVID-END is a CIHR-funded time-limited network of over 50 Canadian and international research groups specializing in evidence synthesis, health technology assessment, and guideline development. COVID-END was created in April 2020 and is partnered with the NCC for Methods and Tools, which is housed at McMaster University in Canada (COVID-END, 2021; Grimshaw et al., 2020; Office of the Chief Science Advisor, 2020a). The COVID-END network performs evidence syntheses (including ‘living’ reviews) and environmental scans on various COVID-19-related topics, such as public health measures, clinical therapeutics, health system resource management, and economic and social responses (COVID-END, 2021; Office of the Chief Science Advisor, 2020a). COVID-END also aims to reduce duplication of research projects by providing a platform for researcher collaboration and coordination (COVID-END, 2021).

## Discussion

In this paper, we described Canada’s science advice ecosystem prior to the COVID-19 pandemic and detailed the new science advisory bodies formed by the federal Health and Science Portfolios throughout the first two years of the COVID-19 pandemic. The COVID-19 pandemic has yielded a significantly more complex federal science advice ecosystem than any previous public health emergencies in Canada. For comparison, one ad-hoc group of volunteer experts (including physicians, infection control practitioners, and administrators) self-organized into a scientific advisory committee during the SARS outbreak in 2003, focusing on quarantine guidelines, as well as hospital isolation precautions, employee screening, and patient transfers (Naylor, 2003). The response to the H1N1 pandemic in 2009 was led by the federal Health Portfolio, with time-limited task groups established to support key actors in the FPT governance structure, such as the PHN Council (Public Health Agency of Canada, 2011). Below, we reflect on our findings and discuss directions for future research to strengthen the federal science advice ecosystem for public health emergencies in Canada.

### Interpretation and implications for science advice for public health emergencies

Canada’s seemingly ad-hoc approach to science advice during the first two years of the COVID-19 pandemic, involving the formation of advisory bodies with unclear coordination and time-limited mandates, presents a concern not only for ongoing pandemic management, but also—as seen in previous outbreaks—sustained preparedness for future public health emergencies. For instance, while the H1N1 pandemic marked significant engagement of mathematical modeling experts to support decisions related to antivirals, vaccines, and other public health measures (Moghadas et al., 2011), new distinct external modeling expert groups were established by both the Health and Science Portfolios during the COVID-19 pandemic (Office of the Chief Science Advisor, 2020c; Public Health Agency of Canada, 2021f, 2022b). An Office of Audit and Evaluation (2020) review of Canada’s response to the first COVID-19 wave further documented that the Health Portfolio lacked a senior lead responsible for modeling and that the coordination between the Health Portfolio and other federal departments involved in modeling was poor (Office of Audit and Evaluation, Health Canada and Public Health Agency of Canada, 2020), potentially resulting in duplicated efforts. Another notable example involves the vaccine-focused advisory groups. Although the federal government has had a longstanding process for obtaining medical and scientific advice on vaccine safety and efficacy through NACI, guidance regarding vaccine distribution was largely the responsibility of PT governments (Ismail et al., 2010). The establishment of the COVID-19 Vaccine Task Force by the Science Portfolio, however, suggests that a logistics-oriented advisory body was needed at the federal level to advise on vaccine procurement, development, and manufacturing (Government of Canada, 2020a, 2020b).

The COVID-19 pandemic can be characterized as a “complex intergovernmental problem”, requiring well-coordinated intergovernmental collaboration (at times through novel avenues) (Paquet and Schertzer, 2020). Wilson et al. (2004) proposed that intergovernmental relationships in public health in Canada can be broadly described by considering vertical (i.e., across the FPT orders of government) and horizontal (i.e., across sectors within each order of government) relationships. Currently, there are no formal horizontal coordination mechanisms for science advice in public health at the federal level, and a 2019 OCSA simulation exercise of science advice in a complex national emergency revealed that science advice was generally fragmented across Canada’s federal departments (Office of the Chief Science Advisor, 2020a). The PHN governance structure and pandemic response plans largely focus on vertical coordination between FPT actors within the health sector. Indeed, the latest (2017) internal operational review of the PHN identified a need to increase the visibility of the PHN among federal departments outside of the Health Portfolio (Dyke, 2017). While the newly established DSA Network is the intended mechanism for horizontal coordination of science advice across federal departments (e.g., through ex-officio membership), it remains to be seen whether this approach has translated to routinized interdepartmental engagement. Notably, among its short- to mid-term goals, the 2021 interim FPT Public Health Response Plan for Ongoing Management of COVID-19 stated that the Health Portfolio intended to work with the OCSA to support the COVID-19 response (Public Health Agency of Canada, 2021f). Explicating this relationship between the Health and Science Portfolios in science advice procurement should be prioritized in Canada’s revised pandemic plans to improve the coordination of science advice across sectors. As recommended by the Senate Committee review of Canada’s H1N1 response, pandemic plans should also be responsive to “[real-time] observations on the ground, rather than a potential worst-case scenario” (Eggleton et al., 2010).

As a stress test of the current systems and processes, the COVID-19 pandemic opens a policy window of opportunity to better institutionalize the federal science advice ecosystem (Di Ruggiero et al., 2022; Kuchenmüller et al., 2022; Paquet and Schertzer, 2020; Quirion et al., 2016). Institutionalization of science advice can be understood as the process and outcome of maintaining and reinforcing norms and practices that, based on collective meaning and endowment of resources, allow expertise and evidence to become legitimized and routinized in health policy-making (Kuchenmüller et al., 2022). Kuchenmüller et al. (2022) emphasize that strengthening the governance of science advice—that is, establishing formalized structures that span the boundaries and promote interaction between science and policy—is one of the foundational domains of institutionalization that protects the science advice ecosystem from contextual and political changes. Resourcing and institutionalizing science advice has also been identified as an important step towards strengthening the governance of public health functions at the federal level overall and achieving learning public health systems in Canada (Di Ruggiero et al., 2022).

Several approaches to institutionalizing science advice for public health emergencies have been proposed in Canada within the past two decades. After SARS, a CIHR-led document review and stakeholder consultation recommended developing a centre of population and public health research evidence, which would function in concert with the then newly established PHAC and NCCs to synthesize, appraise, and disseminate population and public health research and identify evidence gaps (Kiefer et al., 2005). The Fundamental Science Review (2017) recommended legislating an independent National Advisory Council on Research and Innovation, with formalized relationships with the Prime Minister’s Office, the OCSA, and the Ministers of ISED and Health, to oversee the federal science ecosystem, including extramural research funding^10^ (Canada’s Fundamental Science Review and Naylor, 2017). Recently, some scholars have called for creating a federal agency for science advice for health emergencies^11^, solely focused on centralizing and coordinating science advice across federal departments to set the standard for how science advice is procured and used during and outside of health emergencies (Tuohy, 2021).

The need for better institutionalization of well-coordinated intergovernmental collaboration on science advice for public health emergencies is mirrored globally. An International Network for Government Science Advice review of 22 countries’ early COVID-19 responses noted that the predominance of newly struck inter-ministerial committees suggested that countries both recognized a need for better horizontal collaboration and lacked pre-existing mechanisms to implement it (Allen et al., 2020). Another review of 28 countries’ early COVID-19 responses showed that while the formation of multidisciplinary committees was a feature of high-performing responses, poor coordination among such committees to rapidly inform policy decisions was a feature of low-performing responses (Haldane et al., 2021). Recent case studies of the UK, Sweden, Philippines, and North Carolina (US) COVID-19 responses concluded that a standing independent national authority dedicated to science advice for health security and health emergencies was necessary to ensure that pandemic decisions are evidence-informed and expert-driven (Brusselaers et al., 2022; Di Ruggiero et al., 2022; Vallejo and Ong, 2022; Weinkle, 2022). Comparative research is needed to elucidate the optimal institutional arrangement for science advice for public health emergencies in Canada. In particular, we highlight two such research considerations: the independence of science advisory bodies and the role of subnational advisory bodies.

### Directions for future research on science advice in Canada

#### Independence of science advisory bodies

Effective science advice must be, and must be perceived to be, independent from individuals or groups that may have a vested interest in the outcome of the decision for which the advice was requested, such as the decision-makers using the advice (Groux et al., 2018; Royal Society of Canada, 2015). For instance, while the CPHO has a mandate to directly and independently communicate with the Cabinet and the public, some legal scholars have argued that this model of the CPHO role is inherently vulnerable to generating tensions between the (private) advisory role and the (public) communicator role (Fafard et al., 2018). Specifically, the CPHO’s authority to communicate on public health matters is not coupled with security of tenure, which may challenge the CPHO’s ability to openly advocate on issues that run counter to the Cabinet’s position (Fafard et al., 2018; Fierlbeck and Lahey, 2013; MacAulay et al., 2022; Wilson and Keelan, 2008). Similar tensions could be inferred about time-limited expert advisory groups, as, while members of these groups are external to the federal government, the federal departments procuring advice are involved in member selection, establishing the terms of reference, collecting materials to be evaluated by the advisory groups, and terminating participation of individual members or the advisory group overall (Health Canada, 2011; Public Health Agency of Canada, 2021g). Questions have also been raised about the independence of certain arm’s-length advisory bodies, like the CCA. Although the CCA maintains relative autonomy in expert selection, deliberation, and production of their reports, its continued operation depends on federal funding (Groux et al., 2018; Quirion et al., 2016).

Concerns about the independence of science advisory bodies from those requesting advice do not immediately invalidate the advice—indeed, as acknowledged by Bijker et al. (2009), independence and institutionalization of science advisory bodies is a balance, whereby “science that is too detached from the policy domain can barely contribute to the decision process…but science that manifests itself as having a pronounced political message quickly risks becoming a part of political fighting”. Others have similarly argued that proximity of advisory roles, such as the CPHO, to federal actors may give advisors greater influence over the decision-makers’ agenda and enhance the actionability of advice in the context of decision-makers’ priorities (Fafard and Forest, 2016; Groux et al., 2018; MacAulay et al., 2022). Evaluations of the degree of independence of advisory actors (including their means of expert appointment; the flow of information between the advisory groups and decision-makers; and the roles of experts, decision-makers, and commissioners in issue selection and advice generation) (Groux et al., 2018) are needed to inform a nuanced understanding of how advisory group independence shapes science advice in public health emergencies.

Independence of science advisory bodies can also be interpreted as protection of science advice from the influence of commercial or political interests (Groux et al., 2018). The federal policies on external advisory committees state that “a person’s affiliations and interests do not necessarily prevent him or her from being a member of an advisory body, since his or her input could nevertheless be valuable to the advisory body’s mandate” (Health Canada, 2011; Public Health Agency of Canada, 2021g). ISED thus made a deliberate decision to include individuals from the biotechnology sector with potential conflicts of interest (COI) in the COVID-19 Therapeutics (Innovation, Science and Economic Development Canada, 2021d) and Vaccine (Government of Canada, 2020b) Task Forces. Although advisory body members must disclose their interests prior to each meeting and recuse themselves from commenting on issues where they may have real or perceived COIs (Health Canada, 2011; Public Health Agency of Canada, 2021g), the registries of these disclosures only began to be published in September 2020, in response to public pressure (and in breaking from the standard ISED policy for volunteer external advisory groups, whose COIs are not overseen by the federal ethics commissioner and thus, are not listed in the commissioner’s public registry) (Connolly, 2020a, 2020b; Innovation, Science and Economic Development Canada, 2021c; Lexchin, 2022; National Research Council of Canada, 2021a, 2021b). Furthermore, as documented by Lexchin (2022) and Grundy (2022), the nature and severity of these COIs was not publicized and could not be inferred, as the content of Task Force meetings is not publicly available and the primary interest served by the Task Forces is not clearly identified in their mandates. Given the considerable engagement of industry representatives in COVID-19 advisory bodies, it is imperative to evaluate how the present COI mitigation policies have shaped the quality of science advice.

#### Role of subnational authorities in the science advice ecosystem

The presence of PT public health authorities and science advisory structures adds another layer of complexity to the national science advisory milieu. Similar to the role of the CPHO at the federal level, there are inherent contradictions between the advisory and spokesperson functions of CMOHs at the PT level (Fafard et al., 2018; MacAulay et al., 2022). These tensions have become especially salient during the COVID-19 pandemic, as the CMOH scope of work and public visibility have been stretched arguably beyond that which was originally intended (MacAulay et al., 2022). Across Canada’s PTs, this has reignited the debate on the amount of authority CMOHs should have in their advisory, managerial, and public communicator roles, as well as the extent of independence from decision-makers CMOHs should be able to exercise in carrying out these roles (Attaran and Hardcastle, 2020; Bellefontaine, 2020; Weber, 2020). Empirical evaluations of how the CMOH role (and its public and political perception) has evolved throughout the pandemic across different PT institutional and political contexts are ongoing (MacAulay et al., 2022).

Outside the health sector, there were no PT-level science advisory structures analogous to the federal OCSA before the COVID-19 pandemic (Council of Canadian Academies, 2017). The sole exception is the province of Quebec, where the position of the Chief Scientist of Québec was created in 2011 to advise the Minister of the Economy and Innovation and to oversee the three Québec research funds (Gouvernement du Québec, 2016; Quirion et al., 2016). In the first year of the COVID-19 pandemic, the Chief Scientist’s advisory role was extended to supporting the provincial Premier and the Minister of Health and Social Services (Gouvernement du Québec, 2020). New science advisory groups were also created across the other PTs throughout the COVID-19 pandemic. For example, the Ontario COVID-19 Science Advisory Table^12^ is a group of volunteer scientific experts and health system leaders that provides weekly evidence summaries to the COVID-19 Health Coordination Table of the Province of Ontario (Ontario COVID-19 Science Advisory Table, 2022). In British Columbia, the COVID-19 Modeling Group^13^ is a group of experts in epidemiology, mathematics, and data analysis from the province’s three major universities and the private sector, working on rapid response modeling of the COVID-19 pandemic (BC COVID-19 Modeling Group, 2022). Although both groups were largely untethered to the PT governments and formal advisory structures and could be considered “shadow” science advisory actors^14^, their influence on FPT decision-making and the public, and their ability to exercise their independence despite political pressures should be evaluated. It also remains unclear to what extent federal advice was considered in the advice and decisions made at the PT or local level.

### Limitations

A strength of this jurisdictional review is the provision of a comprehensive account of Canada’s federal science advisory ecosystem for public health from SARS to the first two years of the COVID-19 pandemic, with recommendations for policy and research. This complements reviews from other scholars of Canada’s early federal science advice mechanisms not specific to public health (Quirion et al., 2016) and Canada’s federal public health governance structure (Di Ruggiero et al., 2022). Descriptive country-level case studies also serve a critical step for future analyses of cross-country comparisons seeking to understand how contextual differences may have shaped policy outcomes (Marmor, 2017).

Some limitations should also be acknowledged. First, we relied on publicly available literature. As such, while we attempted to achieve a comprehensive search, we are only able to describe the most salient science advice actors and mechanisms documented in the retrieved sources. Relatedly, we did not consider informal pathways to procuring and providing science advice or international exchanges of expertise and evidence. This may have resulted in “false saturation”, whereby certain advisory bodies may have been missed due to their informal status or short lifespan (“survivorship” bias). Second, although most public health measures for COVID-19 are within the remit of subnational authorities in Canada, our focus on science advice at the federal level limits us from commenting on how science advice was mobilized in these subnational structures. Third, the COVID-19 pandemic is still ongoing at the time of writing; thus, our goal was to provide a descriptive account of Canada’s evolving federal science advisory landscape throughout the first two years of the pandemic, rather than to make evaluative inferences about its effectiveness (e.g., in terms of translation of science advice into policy decisions and public guidance, application of science advice in the context of the precautionary principle, or the perceptions of science advisory bodies and their advice by decision-makers and the public). Furthermore, a recent independent assessment of 100 Government of Canada policies found that inferring whether policies were informed by scientific evidence from publicly available information is difficult (Qaiser et al., 2022). This suggests a need for high-quality qualitative research with primary data collection. Finally, while we captured the release of select reports on COVID-19 in certain populations (e.g., long-term care residents, children), given the disproportionate and inequitable impact of the pandemic on elderly, low income, racialized, and migrant groups, an in-depth assessment of the nature and content of the advice provided is needed.

## Conclusion

This detailed review of the first two years of the COVID-19 pandemic in Canada has highlighted the challenges faced by the federal government in procuring science advice to support decision-making during a public health emergency. In its response to COVID-19, the federal government appeared to follow the existing playbook, which was established after the SARS and H1N1 outbreaks and was largely focused on the health sector. However, the COVID-19 pandemic also fueled a rapid proliferation of time-limited science advisory bodies within both the federal Health and Science Portfolios, with limited and unclear mechanisms for horizontal coordination and collaboration. Although the engagement of federal departments outside the health sector is promising, the COVID-19 experience in Canada supports the need to institutionalize science advisory bodies for public health to improve pandemic preparedness and ensure rapid mobilization of well-coordinated and independent advice in future emergencies. This review also identified pressing areas for further inquiry to strengthen science advice for public health in Canada, such as on COI mitigation strategies and the complex interplay between the federal and subnational science advisory bodies.

## Supplementary information

Supplementary material

## Data Availability

Datasets were not generated and/or analyzed for this study. This work analyzed published literature sources that are cited in the manuscript and are accessible either publicly or with academic institutional credentials.

## Footnotes

1 In this paper, when referring to “subnational” governments, we primarily mean PT authorities. However, it is relevant to note that within some PTs, public health governance is further decentralized through regional or municipal health authorities, helmed by locally elected or appointed boards that are led by Medical Officers of Health (Bana et al., 2018; Di Ruggiero et al., 2022). During the COVID-19 pandemic, some of these authorities implemented protective public health measures (e.g., indoor masking) through local bylaws either in conflict with or prior to official provincial guidance.

2 Although largely beyond the scope of the present paper, it should be noted that the three most populous provinces in Canada also have arm’s-length province-wide agencies responsible for public health surveillance, laboratories, and provision of technical advice and guidance—the British Columbia Centre for Disease Control, Public Health Ontario, and l’Institut national de santé publique du Québec (Bana et al., 2018; Di Ruggiero et al., 2022).

3 In Canada, deputy ministers are appointed senior civil servants acting as deputy heads and managerial/administrative heads of FPT governmental departments or ministries. Deputy ministers are primarily responsible for the coordination of policy, program advice, and technical analysis to the minister (the department head—an elected member of Parliament) and for the management of the department and program implementation.

4 The Canadian Ministry—commonly referred to as the Cabinet—consists of the Prime Minister of Canada and 38 federal Ministers. The Cabinet is the central decision-making forum in government that brings all the Ministers together to set the federal government’s policies and priorities. The day-to-day work of the Cabinet is carried out by Cabinet committees, whose mandates and membership are set out by the Prime Minister (with the exception of the Treasury Board, whose mandate and membership are established in law).

5 As outlined in the FPT Public Health Response Plan for Biological Events (Pan-Canadian Public Health Network, 2018b), public health emergency response in Canada is classified into four levels: normal (level 1), heightened (level 2), escalated (level 3), and emergency (level 4). Level 1 or 2 responses typically involve ongoing monitoring and assessment and do not require a coordinated FPT response. Events qualifying for a level 3 or 4 response typically affect multiple jurisdictions and require federal surge capacity or centralized planning; bulk purchasing and consistent use of limited resources (e.g., medical countermeasures); new or revised technical guidance documents and recommendations; collated national epidemiological data; and consistent approaches to border screening, contact identification and follow-up, and public and professional communications (Pan-Canadian Public Health Network, 2018b). Level 3 or 4 events thus trigger the implementation of a coordinated FPT response and activation of the SAC and the SAC Secretariat (Pan-Canadian Public Health Network, 2018b).

6 In May 2021, the unanimous adoption of Motion M-38 marked the creation of a new Standing Committee on Science and Research in the House of Commons, effective in the next Parliament (Dufour, 2021). The Standing Committee was created to affirm the government’s commitment to evidence-informed decision-making (Evidence for Democracy, 2021) and its mandate includes “all matters relating to science and research, including any reports of the Chief Science Advisor” (House of Commons Canada, 2021).

7 As we focus on describing the emergence of new science advisory bodies, a detailed discussion of the federal whole-of-government incident management decision-making structure is largely beyond the scope of this paper. Nonetheless, the apparent need for a federal decision-making committee outside of the existing incident response mechanism is notable. The Sub-Committee on the Federal Response to COVID-19 is situated within the Cabinet Committee on Safety, Security and Emergencies, is chaired by the Minister of Intergovernmental Affairs, Infrastructure and Communities, and has representation from 11 federal ministers (Prime Minister of Canada Office, 2021c). The Cabinet Committee on Safety, Security and Emergencies, chaired by the Minister of Emergency Preparedness, considers national safety and security threats, manages ongoing emergencies, and ensures strategic and integrated emergency management across the federal government (Prime Minister of Canada Office, 2021a).

8 The Public Health Working Group on Remote and Isolated Communities is a new group established during the COVID-19 pandemic in response to the recommendations of the 2017 internal operational review of the PHN, which identified a need for better engagement of remote and Indigenous communities in the PHN structure (Di Ruggiero et al., 2022; Dyke, 2017). The Surveillance Expert Working Group and the Canadian Pandemic Influenza Plan Task Group are existing PHN task groups, named in the 2018 FPT Public Health Response Plan for Biological Events (Pan-Canadian Public Health Network, 2018b).

9 The overall objective for Canada’s pandemic preparedness and response—“first, to minimize serious illness and overall deaths, and second to minimize societal disruption among Canadians as a result of an influenza pandemic”—has remained unchanged from the first release of the CPIP in 2004 (Pan-Canadian Public Health Network, 2018a).

10 Progress on this recommendation is ongoing, with ISED announcing the Council on Science and Innovation in January 2019; however, the status of the Council is not publicly known as of December 2021 (Canada’s Fundamental Science Review & Naylor, 2017; Innovation, Science and Economic Development Canada, 2019).

11 Some scholars have also called for turning the new Cabinet Sub-Committee on the Federal Response to COVID-19 into a standing committee on emergencies to ensure that emergency planning is an ongoing function between acute crises (Cappe, 2020). Formalizing the relationship between the science advice ecosystem with this decision-making body is an important consideration for institutionalizing science advice for public health in Canada.

12 At the time of writing, the Ontario COVID-19 Science Table was housed at the Dalla Lana School of Public Health at the University of Toronto and its Scientific Director and Secretariat were funded by the Dalla Lana School of Public Health and Public Health Ontario (Ontario COVID-19 Science Advisory Table, 2022). The Science Table’s outputs (e.g., briefings, modeling projections, and other reports) were publicly available.

13 At the time of writing, the COVID-19 Modeling Group was sponsored by the participating provincial universities and the Pacific Institute for the Mathematical Sciences (a mathematics research consortium of select universities from Canada and the United States) (BC COVID-19 Modeling Group, 2022). The Modeling Group’s outputs were publicly available.

14 Shadow science advice can be defined as “formal or informal mechanisms of advice established outside of governmental science advisory processes to provide a counter or opposition body of legitimate, authoritative, and credible guidance to policy makers” (Pielke, 2021). Several shadow advisory groups have emerged during the COVID-19 pandemic internationally, including the Independent SAGE in the UK, the Science Forum COVID-19 in Sweden, the Red Team in the Netherlands, and the OCTA Research Group in the Philippines (Brusselaers et al., 2022; Pielke, 2021; Vallejo & Ong, 2022). Many such groups have gained legitimacy in the public eye due to their direct communication with the media and the public; however, the ethical and appropriate practices for shadow science advice remain to be established (Pielke, 2021). Interestingly, according to a March 31, 2022 announcement, the Ontario COVID-19 Science Table (a volunteer group of experts previously untethered to the provincial government) has begun to be folded into Public Health Ontario—an arm’s-length governmental agency (Wallace, 2022).
