# Supplementary material for: Mobilization of science advice by the Canadian federal government to support the COVID-19 pandemic response
Source: Humanit Soc Sci Commun. 2023 Jan 17;10(1):19. doi: 10.1057/s41599-023-01501-8 (PMC9844194; doi:10.1057/s41599-023-01501-8)
Supplement: Supplementary file 1 — Supplementary material [file 41599_2023_1501_MOESM1_ESM.docx]

**Supplementary material**

**Mobilization of science advice by the Canadian federal government to support the COVID-19 pandemic response**

**Authors:**

Dominika Bhatia^1,2^*, [dominika.bhatia@mail.utoronto.ca](mailto:dominika.bhatia@mail.utoronto.ca)

Sara Allin^1,3^*^§^, [sara.allin@utoronto.ca](mailto:sara.allin@utoronto.ca)

Erica Di Ruggiero^3-5^*^§^, [e.diruggiero@utoronto.ca](mailto:e.diruggiero@utoronto.ca)

**Author affiliations:**

^1^ North American Observatory on Health Systems and Policies, Toronto, Ontario, Canada

^2^ Women’s College Research Institute, Women’s College Hospital, Toronto, Ontario, Canada

^3^ Institute of Health Policy, Management and Evaluation, Dalla Lana School of Public Health, University of Toronto, Toronto, Ontario, Canada

^4^ Social and Behavioural Health Sciences Division, Dalla Lana School of Public Health, University of Toronto, Toronto, Ontario, Canada

^5^ Centre for Global Health Dalla Lana School of Public Health, University of Toronto, Toronto, Ontario, Canada

* All authors are corresponding authors

^§^ Senior co-authors

**Supplementary file 1.** Extended literature review methodology

This paper presents a jurisdictional case study of Canada’s federal science advice ecosystem, reliant on a literature review of publicly accessible primary (i.e., Government of Canada technical reports and guidance) and secondary (i.e., peer-reviewed literature) documents. This study was conducted as part of an international case series – Evaluation of Science Advice in a Pandemic Emergency (EScAPE, <https://escapecovid19.org/>) – comprising 22 jurisdictional case studies aimed at establishing a baseline understanding of jurisdiction-level science advisory processes in a pandemic context. To enable future comparative cross-case analyses, a common case study framework was developed by the EScAPE investigators. This framework informed our document collection process and synthesis, as well as the structure of the present paper.

Specifically, the following steps were followed, as set out in the framework:

1. Documentation of the national-level pre-COVID-19 science advisory ecosystem (the pandemic “playbook”);
2. Development of a chronological narrative of when the pre-COVID-19 science advisory bodies and mechanisms were created and in what context;
3. Documentation of the science advisory ecosystem during the COVID-19 pandemic (including mobilization or modification of existing bodies and creation of new ones);
4. Comparison of the science advisory ecosystem that emerged during the COVID-19 pandemic with the initial playbook.

**Definitions**

We defined the concepts of science advice, science advisory body, science advice ecosystem, scientific experts, and scientific evidence in line with established definitions in peer-reviewed literature, as outlined in **Table 1**.

**Table 1.** Definitions of key concepts

| **Concept** | **Definition** |
| --- | --- |
| Science advice | The process, structures, and institutions through which governments and decision-makers receive and consider science and technology inputs to public policy development (Quirion et al., 2016). |
| Science advisory body | Any body that, when requested, provides scientific advice to government as one of its main, although not necessarily only, functions (Groux et al., 2018). |
| Science advice ecosystem | All science advisory bodies involved in evidence generation (including primary data collection and evidence synthesis), knowledge brokerage (i.e., the intermediary function of providing policy options to decision-makers based on evidentiary input), and science communication to the public (Gluckman et al., 2021; Groux et al., 2018; Organization for Economic Co-operation and Development, 2015; Stewart et al., 2019).  An individual science advisory body may have any combination of these functions, with some also holding decision-making authority (Global Commission on Evidence to Address Societal Challenges, 2022; Stewart et al., 2019). |
| Scientific expert | Individual with in-depth knowledge about a particular field, with said knowledge validated through qualifications, certification, peer recognition, or other accepted credentialing methods (Global Commission on Evidence to Address Societal Challenges, 2022; Owens, 2018). |
| Scientific evidence | Findings from research and other forms of knowledge (Global Commission on Evidence to Address Societal Challenges, 2022; Office of the Prime Minister’s Science Advisory Committee & Gluckman, 2013; World Health Organization, Regional Office for Europe, 2022). |

**Search strategy**

Based on the definitions provided above, the government science advice ecosystem may involve intramural government research units, arm’s-length academic organizations, permanent or time-limited expert advisory committees, science and technology councils, and formal advisors to heads of government or legislators (Gluckman et al., 2021; Office of the Prime Minister’s Science Advisory Committee & Gluckman, 2013; Organization for Economic Co-operation and Development, 2015). A traditional systematic search of bibliographic databases is not well-positioned to retrieve primary sources on science advisory bodies. For this reason, we searched Government of Canada websites for pandemic emergency management plans, evaluations of responses to previous infectious disease emergencies (i.e., severe acute respiratory syndrome [SARS] and H1N1), general annual reports of governmental agencies’ activities, and lists of and reports generated by advisory committees to governmental agencies (see examples in **Table 2**).

**Table 2**. Examples of primary sources searched

| **Type** | **Example** |
| --- | --- |
| Pandemic emergency plan | Canadian Pandemic Influenza Preparedness: Planning Guidance for the Health Sector^[[1]](#footnote-1)^ |
| Evaluation of previous pandemic response | Senate Report on Canada’s Response to the 2009 H1N1 Influenza Pandemic^[[2]](#footnote-2)^ |
| Government agency website | Pan-Canadian Public Health Network^[[3]](#footnote-3)^ |
| General annual report of government agency | Chief Science Advisor Annual Report 2019-20^[[4]](#footnote-4)^ |
| List of advisory committees to government agency | List of external advisory bodies for the Public Health Agency of Canada^[[5]](#footnote-5)^ |

We considered all document versions^[[6]](#footnote-6)^ (e.g., revisions to pandemic plans after H1N1 and throughout the COVID-19 pandemic). Primary document searches were supplemented by searches for peer-reviewed articles in ProQuest and Google Scholar, using combinations of terms related to the Canadian setting and government agencies, science, emergency management, public safety, public health, and pandemics. Reference lists of primary and secondary documents were reviewed to retrieve additional sources in an iterative snowball fashion.

The document search was focused on the period between the 2003 SARS outbreak in Canada and December 2021. The starting bound of this time period was selected because the SARS outbreak, which affected Canada more than any other country outside of Asia (where the virus was originally identified), triggered crucial recommendations and reforms that shaped Canada’s federal public health science advice landscape (e.g., through the introduction of the national public health agency) (Naylor, 2003). The ending bound of this time period enabled us to capture the immediate effects of the COVID-19 pandemic on the federal science advice ecosystem^[[7]](#footnote-7)^.

**Selection criteria**

We identified key science advisory bodies involved in providing guidance to federal agencies, considering all types of science advisory bodies, including those involved in evidence generation, brokerage, communication, and decision-making. We reviewed advisory body mandates, terms of reference, membership lists, and outputs. To be considered part of the federal science advice ecosystem, science advisory bodies had to demonstrate evidence of an advisory relationship^[[8]](#footnote-8)^ with federal actors (e.g., being mandated to advise a federal agency or being included among listed federal advisors or partners).

**Document review completeness check with local experts**

The preliminary findings of the literature review were shared in November 2021 with nine local experts via email or conference call to ensure that relevant science advisory bodies were not missed. The preliminary findings (available from the authors on reasonable request) shared with the local experts included an overview of key federal science advisory bodies established prior to (e.g., federal Health and Science Portfolios) and during (e.g., time-limited expert advisory groups) the COVID-19 pandemic. Local experts were mid- to senior-level academics and public servants employed by policy, non-governmental, and academic public health organizations in Canada. These individuals were selected because they either had “outsider” (e.g., through involvement in research in this area) or “insider” (e.g., through participation in the provision or soliciting of science advice in their past or current roles) knowledge of science advice for public health in Canada.

The local experts were asked the following questions:

- Do these preliminary findings resonate with you and your knowledge of federal science advice mechanisms used during COVID-19?
- Have we missed anything (e.g., mechanisms for soliciting or coordinating science advice across government agencies)?
- What are your takeaways from these findings?

The purpose of local expert engagement was to check the completeness of the findings emerging from the literature review, rather than to generate additional data; as such, the local experts are not identified in this paper. Based on local expert feedback, we performed additional literature searches to fill in any identified gaps.

**Document review synthesis and manuscript structure**

To address our objective of documenting the mobilization and evolution of Canada’s federal science advice ecosystem during the first two years of the COVID-19 pandemic, we synthesized our findings descriptively, with a focus on delineating the actors involved in providing advice and any relationships between them. A descriptive focus is reasonable because descriptive country-level case studies serve a critical step for future analyses of science advice effectiveness within the jurisdiction, as well as for cross-country comparisons seeking to understand how contextual differences may have shaped policy outcomes (Marmor, 2017). As our focus is descriptive rather than evaluative, theoretical and conceptual literatures on policy advice and procuring advice are not directly engaged here. The manuscript structure was informed by the EScAPE framework, first presenting the federal public health science advice playbook and then highlighting new advisory bodies created for convening experts and generating evidence to inform decision-making (see **Table 1** for definitions).

**References**

Global Commission on Evidence to Address Societal Challenges. (2022). *The Evidence Commission report: A wake-up call and path forward for decision-makers, evidence intermediaries, and impact-oriented evidence producers* (p. 144). McMaster Health Forum. https://www.mcmasterforum.org/networks/evidence-commission/report/english

Gluckman, P. D., Bardsley, A., & Kaiser, M. (2021). Brokerage at the science–policy interface: From conceptual framework to practical guidance. *Humanities and Social Sciences Communications*, *8*(1), 84. https://doi.org/10.1057/s41599-021-00756-3

Groux, G. M. N., Hoffman, S. J., & Ottersen, T. (2018). A Typology of Scientific Advisory Committees. *Global Challenges*, *2*(9), 1800004. https://doi.org/10.1002/gch2.201800004

Marmor, T. R. (2017). Comparative Studies and the Drawing of Policy Lessons: Describing, Explaining, Evaluating, and Predicting. *Journal of Comparative Policy Analysis: Research and Practice*, *19*(4), 313–326. https://doi.org/10.1080/13876988.2017.1279439

Naylor, C. D. (2003). *Learning from SARS: Renewal of public health in Canada: a report of the National Advisory Committee on SARS and Public Health*. National Advisory Committee on SARS and Public Health. https://www.canada.ca/en/public-health/services/reports-publications/learning-sars-renewal-public-health-canada.html

Office of the Prime Minister’s Science Advisory Committee, & Gluckman, P. D. (2013). *The role of evidence in policy formation and implementation: A report from the Prime Minister’s Chief Science Advisor*. Office of the Prime Minister’s Science Advisory Committee.

Organization for Economic Co-operation and Development. (2015). *Scientific Advice for Policy Making: The Role and Responsibility of Expert Bodies and Individual Scientists* (OECD Science, Technology and Industry Policy Papers No. 21; OECD Science, Technology and Industry Policy Papers, Vol. 21). https://doi.org/10.1787/5js33l1jcpwb-en

Owens, S. (2018). Trust in Experts? Knowledge, Advice and Influence in Environmental Policy. *Science in Times of Challenged Trust and Expertise*, 10–19.

Quirion, R., Carty, A., Dufour, P., & Jabr, R. (2016). Reflections on science advisory systems in Canada. *Palgrave Communications*, *2*(1), 16048. https://doi.org/10.1057/palcomms.2016.48

Stewart, R., Dayal, H., Langer, L., & van Rooyen, C. (2019). The evidence ecosystem in South Africa: Growing resilience and institutionalisation of evidence use. *Palgrave Communications*, *5*(1), 90. https://doi.org/10.1057/s41599-019-0303-0

World Health Organization, Regional Office for Europe. (2022). *Defining evidence*. World Health Organization. https://www.euro.who.int/en/data-and-evidence/evidence-informed-policy-making/about-us

1. <https://www.canada.ca/en/public-health/services/flu-influenza/canadian-pandemic-influenza-preparedness-planning-guidance-health-sector.html> [↑](#footnote-ref-1)
2. <https://sencanada.ca/content/sen/Committee/403/soci/rep/rep15dec10-e.pdf> [↑](#footnote-ref-2)
3. <https://www.phn-rsp.ca/en/index.html> [↑](#footnote-ref-3)
4. <https://science.gc.ca/site/science/en/office-chief-science-advisor/annual-reports/chief-science-advisor-annual-report-2019-20> [↑](#footnote-ref-4)
5. <https://www.canada.ca/en/public-health/corporate/mandate/about-agency/external-advisory-bodies/list.html> [↑](#footnote-ref-5)
6. Any archived document versions were retrieved using the Wayback Machine website (<https://archive.org/web/>). [↑](#footnote-ref-6)
7. The start of 2022 was accompanied by the introduction of a new variant of concern (Omicron) and significant changes in Canada’s pandemic response, with eventual suspension of widespread testing and public reporting on COVID-19 epidemiology, as well as removal of public health COVID-19 containment measures across Canadian jurisdictions (for a detailed timeline of public health interventions implemented for COVID-19 in Canada, see: <https://www.cihi.ca/en/canadian-covid-19-intervention-timeline>). In addition, certain ad-hoc science advice bodies with time-limited mandates began to be absorbed into existing governmental institutions, changing their governance and accountability structure (e.g., Ontario COVID-19 Science Table). This suggests that the year 2022 may have marked a new phase of science advice for public health in Canada, which requires separate focused study. [↑](#footnote-ref-7)
8. It should be noted that while federal funding agencies were included in the federal science advisory ecosystem, we considered the discussion of specific federal funding calls for external COVID-19 research as a potential science advisory mechanism to be outside the scope of this paper. The objectives and timelines of external research studies are largely shaped by independent academic researchers; furthermore, it may not be possible to reliably infer from publicly available information whether and to what extent (i) governmental actors may be involved as knowledge users throughout the research process; and (ii) research findings may be shared with governmental actors to inform decision-making. [↑](#footnote-ref-8)
